# Analysis of Common and Specific Mechanisms of Liver Function Affected by Nitrotoluene Compounds

**DOI:** 10.1371/journal.pone.0014662

**Published:** 2011-02-08

**Authors:** Youping Deng, Sharon A. Meyer, Xin Guan, Barbara Lynn Escalon, Junmei Ai, Mitchell S. Wilbanks, Ruth Welti, Natàlia Garcia-Reyero, Edward J. Perkins

**Affiliations:** 1 Department of Internal Medicine, Rush University Cancer Center, Rush University Medical Center, Chicago, Illinois, United States of America; 2 University of Louisiana at Monroe, Monroe, Louisiana, United States of America; 3 SpecProc Inc., Vicksburg, Mississippi, United States of America; 4 US Army Engineer Research and Development Center, Vicksburg, Mississippi, United States of America; 5 School of Computing, University of Southern Mississippi, Hattiesburg, Mississippi, United States of America; 6 Division of Biological Sciences, Kansas State University, Manhattan, Kansas, United States of America; 7 Jackson State University, Jackson, Mississippi, United States of America; Indiana University, United States of America

## Abstract

**Background:**

Nitrotoluenes are widely used chemical manufacturing and munitions applications. This group of chemicals has been shown to cause a range of effects from anemia and hypercholesterolemia to testicular atrophy. We have examined the molecular and functional effects of five different, but structurally related, nitrotoluenes on using an integrative systems biology approach to gain insight into common and disparate mechanisms underlying effects caused by these chemicals.

**Methodology/Principal Findings:**

Sprague-Dawley female rats were exposed via gavage to one of five concentrations of one of five nitrotoluenes [2,4,6-trinitrotoluene (TNT), 2-amino-4,6-dinitrotoluene (2ADNT) 4-amino-2,6-dinitrotoulene (4ADNT), 2,4-dinitrotoluene (2,4DNT) and 2,6-dinitrotoluene (2,6DNT)] with necropsy and tissue collection at 24 or 48 h. Gene expression profile results correlated well with clinical data and liver histopathology that lead to the concept that hematotoxicity was followed by hepatotoxicity. Overall, 2,4DNT, 2,6DNT and TNT had stronger effects than 2ADNT and 4ADNT. Common functional terms, gene expression patterns, pathways and networks were regulated across all nitrotoluenes. These pathways included NRF2-mediated oxidative stress response, aryl hydrocarbon receptor signaling, LPS/IL-1 mediated inhibition of RXR function, xenobiotic metabolism signaling and metabolism of xenobiotics by cytochrome P450. One biological process common to all compounds, lipid metabolism, was found to be impacted both at the transcriptional and lipid production level.

**Conclusions/Significance:**

A systems biology strategy was used to identify biochemical pathways affected by five nitroaromatic compounds and to integrate data that tie biochemical alterations to pathological changes. An integrative graphical network model was constructed by combining genomic, gene pathway, lipidomic, and physiological endpoint results to better understand mechanisms of liver toxicity and physiological endpoints affected by these compounds.

## Introduction

Nitro-reduction of the munitions compound 2,4,6-trinitrotoluene (TNT) during environmental degradation produces 2-amino-4,6-dinitrotoluene (2A-4,6DNT or 2ADNT) and 4-amino-2,6 dinitrotoluene (and 4A-2,6DNT or 4ADNT). Other structurally similar munitions compounds include the dinitrotoluenes, 2,4-dinitrotoluene (2,4DNT) and its isomer 2,6-dinitrotoluene (2,6DNT). This class of chemicals has been reported to contaminate land, water and retired ammunitions plants as a result of military activities and a series of manufacturing processes [1]–[3]. The major biological effects of TNT subchronic and chronic exposure are anemia (both aplastic and hemolytic), methemaglobinemia, hypercholesterolemia, and testicular atrophy with accompanying histological lesions [4]. DNT and ADNT exposure have been reported to cause hepatomegaly and splenomegaly in addition to cytotoxic and genotoxic effects [5]–[8]. However, the mutagenicity of TNT is controversial [9]. TNT has been shown to interact with hemoglobin to form adducts, mainly 2ADNT and 4ADNT, while 2,4DNT and 2,6DNT have been reported to produce 4-amino-2-nitrotoluene (4A2NT) and 2,4-toluenediamine (24TDA), and 2-amino-6-nitrotoluene (2A6NT) and 26TDA, respectively [10], [11].

Fathead minnow exhibited that lipid metabolism and oxygen transport pathways were the primary mechanism for 2,4DNT induced toxicity in fish [2]. The exposure of *Eisenia fetida* modulated the expression of genes involved in multiple biological processes including muscle contraction, neuronal signaling and growth, ubiquitinylation, fibrinolysis and coagulation, iron and calcium homeostasis, oxygen transport, and immunity [12].

Although many physiological and toxic responses of these compounds are known, a systematic study using toxicogenomics along with such responses is warranted in order to fully understand the relative toxicity of these nitroaromatics in mammals. Moreover, the underlying mechanisms of toxicity induced by these compounds especially for acute exposures, are largely unknown, and little if any toxicogenomics and systems biology studies have been conducted in exposed mammals. An acute exposure is advantageous in that early biomarkers and pathways may be identified to predict future toxicity induced by these chemicals.

To facilitate study of mechanisms, we chose to profile and integrate genomics, system biology and classical toxicological endpoints from the same biological samples shortly after a single exposure to one of five concentrations of one of the 5 nitrotoluene compounds with sacrifice and tissue collection after 24 or 48 hours. Assessment included physiological endpoint measurements and genomics of liver tissues using microarrays. Overall, we found that the expression results correlated well with toxicological and pathological results. Although each compound affected a distinctive expression pattern, a common group of genes and pathways were significantly affected by most of the compounds. We demonstrate a network mode of action based on the gene expression profiles, which could explain why the physiological responses occurred after exposure to these compounds.

## Results

### Analytical chemistry

Blood from rats exposed to each of the five nitrotoluenes, TNT, 2ADNT, 4ADNT, 2,6DNT, and 2,4DNT, for either 24 or 48 h was examined for the presence of parent compound and/or possible nitrotoluene metabolites using HPLC. At 24 h, measurable concentrations of nitrotoluenes were found only in blood of the two highest TNT dosed and 3 highest dosed 2ADNT dosed animals (File S1). 2ADNT was the only metabolite observed in blood of the two highest dosed TNT treated animals 24 h after exposure. No measurable amounts of any of the five nitrotoluene compounds or their degradation products were found in blood 48 h after the exposure.

Liver tissue of exposed and control rats were examined for the presence of parent chemicals and their metabolites 24 and 48 h after exposure. In livers of exposed rats, only the parent compounds TNT, 2ADNT, and 2,4DNT were detected at 24 h, but only in one replicate (File S1). Metabolites of TNT were observed in livers of 192 and 384 mg/kg TNT exposed rats at 24 h after exposure where 2,6-diamino-4-nitrotoluene and 2,4-diamino-6-nitrotoluene were detected.

At lower doses of 96 and 48 mg/kg TNT-treatments, 2ADNT was found in liver of one animal for each dose, while no 4ADNT was observed. No measurable concentrations of the other four nitrotoluene parent compounds were found in livers of animals after 48 h (File S1). Further metabolic products such as azoxytoluenes and benzylalcohols were not measured under the protocol utilized.

### Toxicity measurements

Although the upper limit of chemical dose ranges were designed to be one-half the published acute oral LD_50_, lethality was observed within the dose range used for the dinitrotoluenes. The TNT dose was based on the publication of Reddy at al., [13]. The doses of 2,4DNT and 2,6DNT were based on the publication of Ellis et al.,[14]. The dose selection for 2ADNT and 4ADNT were based on the report of Ellis et al., [15].

All treated rats expired at the high dose (398 mg/kg) of 2,6DNT. The high dose of 2,4DNT caused deaths of 2 of 5 and 1 of 5 rats at 24 and 48 h, respectively. Systemic toxicity was also indicated for all compounds by decreases in body weight gain over the 48 h observation period. Body weight gain for vehicle-treated rats was 17±1 g (mean±SEM, n = 5) compared to a loss of 6−7 g for 199 mg/kg 2,6DNT and high dose (384 mg/kg) TNT. Weight gains were reduced to between 1 to 4 g for rats treated with 50 and 99 mg/kg 2,6DNT, 398 mg/kg 2,4DNT and 192 mg/kg TNT and to 5−9 g for 48 and 96 mg/kg TNT, 174 and 348 mg/kg 2ADNT, and 374 mg/kg 4ADNT. Body weight loss associated with higher doses of TNT and dinitrotoluenes was possibly related to reduced food consumption as indicated by a coincident decrease in serum alkaline phosphatase (AlkP, Tables 1,2) [16].

**Table 1:** Clinical Chemistry Outcomes Affected by 2,4- and 2,6-DNT and TNT^a^.

Dose	Time	Albumin	ALT^b^	AST	AlkP	Glucose	Na
mg/kg	hr	g/dL	IU/L	IU/L	IU/L	mg/dL	mmol/L
				**24DNT**			
0	24	1.38±0.04	132.2±2.9	237.2±33.9	273.2±29.5	226.6±14.9	143.8±0.8
	48	1.58±0.02	104.6±6.6	311.8±65.8	273.0±21.4	260.8±25.0	143.4±0.7
5	24	1.42±0.04	128.8±3.7	363.8±50.7	269.0±20.2	255.4±11.3	145.0±1.2
	48	1.56±0.02	95.0±5.0	275.2±58.1	262.0±15.5	241.4±22.4	144.5±0.7
50	24	1.42±0.04	139.4±12.0	384.4±87.9	310.6±26.8	227.2±14.3	143.6±0.7
	48	1.54±0.04	95.2±11.1	316.4±111.4	230.0±21.0	219.4±8.8	144.4±0.8
99	24	1.30±0.03	124.6±15.3	506.0±111.4	279.0±36.2	234.8±19.5	140.8±3.8
	48	1.38±0.05**	97.2±4.1	280.4±41.0	224.6±28.8	221.6±12.3	143.2±0.9
198	24	1.36±0.05	96.0±21.5	392.4±86.5	237.6±15.6	225.4±15.1	139.2±2.6
	48	0.90±0.06**	96.0±1.3	440.0±35.4	112.5±24.3**	393.5±93.2	137.0±1.9**
398	24	1.5, 1.4	116, 60	514, 200	115, 95	184, 275	142, 139
	48	0.77±0.03**	132.3±13.0	755.0±62.6**	130.0±7.5**	686.7±48.2**	127.7±2.5**
				**26DNT**			
0	24	1.46±0.04	144.4±4.2	332.0±32.3	277.4±19.0	257.8±12.7	145.2±0.5
	48	1.54±0.06	98.4±5.6	154.2±12.2	250.2±5.1	221.3±9.4	144.0±2.0
5	24	1.54±0.02	128.8±9.8	306.0±47.3	318.8±17.1	226.0±21.2	147.8±1.0
	48	1.64±0.02	98.2±7.9	161.4±8.4	258.0±12.9	255.0±2.0	147.0±1.0
25	24	1.52±0.02	163.6±16.8	567.4±137.3	334.6±22.1	214.2±24.0	145.0±0.8
	48	1.62±0.03	95.4±5.0	267.2±48.9	279.0±27.4	211.6±8.6	146.4±0.4
50	24	1.54±0.02	138.2±7.1	341.8±36.7	312.6±22.3	246.4±13.2	145.4±0.8
	48	1.42±0.05	**738.8±627.7**	**1529.0±1310.6**	257.8±15.9	224.0±11.0	144.0±2.0
99	24	1.46±0.05	174.8±27.7	633.6±164.7	241.6±18.0	193.6±11.1*	141.8±1.8
	48	1.30±0.14	**743.0±622.3**	**1046.6±697.6**	269.0±29.4	186.0±12.1	145.0±0.6
199	24	1.54±0.04	150.0±27.9	779.0±176.2*	199.4±27.8	293.4±10.6	136.6±0.9**
	48	1.0	208	234	128	nd	nd
				**TNT**			
0	24	1.38±0.02	140.4±33.0	366.8±52.6	294.2±13.7	174.4±38.5	151.6±6.6
	48	1.52±0.04	172.4±24.1	463.3±145.0	252.8±36.9	222.0±20.4	141.8±1.2
5	24	1.34±0.02	105.0±8.5	309.6±44.9	347.0±13.9	217.2±9.3	146.2±0.4
	48	1.58±0.02	147.8±40.0	267.5±53.3	222.0±27.5	246.6±16.0	144.4±0.7
48	24	1.40±0.05	81.6±6.5	329.8±51.4	318.0±10.2	209.2±9.5	145.4±0.9
	48	1.60±0.03	129.8±5.8	478.2±90.6	247.2±16.9	232.4±19.3	143.0±1.1
96	24	1.34±0.02	76.0±15.9*	408.8±108.6	284.2±11.7	208.8±9.4	141.8±1.4
	48	1.50±0.03	125.0±18.7	433.8±103.6	190.6±21.0	224.4±14.9	144.6±1.2
192	24	1.40±0.03	45.6±6.8**	251.2±53.0	272.2±28.0	260.2±18.3	141.6±1.1
	48	1.36±0.05*	128.4±53.0	475.2±143.8	215.0±20.1	239.4±15.3	136.8±3.4
384	24	1.42±0.04	42.4±5.8**	248.2±48.8	224.4±20.9*	228.4±42.9	141.8±1.4
	48	1.33±0.02**	122.5±55.3	354.9±68.7	208.0±13.2	211.8±13.2	132.8±1.1**

a Values are means±SEM for n = 5, except for 198 and 398 mg/kg 24DNT 48 h where n = 3 and for 398 mg/kg 24DNT 24 h where individual values are shown. For 199 mg/kg 26DNT 48 h values are from pooled sample for 3 rats. Means that differed from concurrent vehicle controls (0 mg/kg) are indicated by * or ** for p<0.05 or 0.01, respectively.

b Abbreviations. AlkP = alkaline phosphatase, ALT and AST = alanine and aspartate aminotransferase, resp., nd = not determined.

**Table 2:** Clinical Chemistry Outcomes Affected by 2ADNT and 4ADNT^a^.

Dose	Time	Albumin	ALT^b^	AST	AlkP	Glucose	Na
mg/kg	hr	g/dL	IU/L	IU/L	IU/L	mg/dL	mmol/L
				**2ADNT**			
0	24	1.42±0.04	116.8±4.2	314.7±72.0	359.6±20.4	224.8±15.7	143.6±0.8
	48	1.60±0.04	138.0±7.1	225.6±32.6	283.8±43.7	253.6±9.4	141.4±1.1
4	24	1.36±0.02	151.2±41.1	403.8±112.1	328.4±11.0	238.2±12.9	141.6±1.4
	48	1.52±0.04	143.4±21.0	364.8±105.8	204.6±29.0	200.8±6.6*	141.6±1.7
44	24	1.36±0.04	92.0±2.5	211.6±11.1	387.8±30.9	228.2±3.4	142.8±0.4
	48	1.58±0.07	174.6±35.0	477.4±107.7	203.4±19.4	217.0±15.8	139.8±2.1
87	24	1.38±0.04	91.6±6.1	341.8±58.0	338.4±28.9	221.0±5.5	141.8±0.4
	48	1.56±0.05	99.4±4.4	128.8±10.1	205.8±27.7	212.2±8.4	144.6±0.4
174	24	1.34±0.02	91.6±2.8	316.2±53.4	249.0±24.7	207.0±10.4	142.0±0.8
	48	1.60±0.03	109.6±7.1	166.0±13.5	230.2±11.3	226.4±16.9	143.6±0.4
348	24	1.34±0.05	89.4±9.6	352.8±44.1	388.0±13.2	241.6±14.8	139.8±0.9*
	48	1.52±0.04	127.4±20.4	268.4±124.5	212.0±19.4	213.0±14.9	141.6±1.3
				**4ADNT**			
0	24	1.30±0.01	143.2±6.6	417.4±37.8	342.8±33.3	284.4±11.8	143.6±0.8
	48	1.42±0.04	195.2±18.9	630.8±76.4	216.8±23.2	232.4±11.0	136.2±0.5
5	24	1.34±0.02	122.6±6.1	364.4±65.7	329.0±19.9	235.2±6.8	144.8±0.4
	48	1.42±0.02	202.4±43.5	489.8±161.9	238.0±21.1	253.4±16.0	136.6±1.4
47	24	1.32±0.04	86.6±4.7**	201.0±27.7**	339.6±25.5	225.0±11.9**	145.0±0.5
	48	1.50±0.04	254.6±49.0	733.8±150.0	307.0±20.7	243.6±16.0	134.4±1.2
94	24	1.32±0.04	66.6±3.4**	182.2±14.6**	342.6±19.7	226.0±13.3**	144.6±0.5
	48	1.46±0.02	727.2±253.5*	1911.8±421.0*	280.0±33.7	324.4±32.9**	126.2±2.6**
187	24	1.32±0.02	56.4±6.6**	243.6±37.8*	323.2±35.2	202.8±5.3**	143.4±0.9
	48	1.42±0.06	405.0±57.4	1505.8±228.2	205.8±11.9	275.6±13.9	130.4±1.8
374	24	1.42±0.02*	48.2±4.9**	283.4±26.7	317.4±34.4	230.6±8.7**	142.2±1.2
	48	1.40±0.3	271.0±95.0	1247.4±305.4	377.8±124.6	260.8±16.4	131.8±1.7

a Values are means±SEM for n = 5. Means that differed from concurrent vehicle controls (0mg/kg) are indicated by * or ** for p<0.05 or 0.01, respectively.

b Abbreviations. AlkP = alkaline phosphatase, ALT and AST = alanine and aspartate aminotransferase, resp.

Additional differences detected from the comprehensive serum metabolic panel included effects on serum albumin, total protein, glucose and Na (Tables 1,2). Serum albumin was the parameter changed at the lowest level of exposure also known as lowest observed effect level (LOEL). Serum albumin decreased at 48 h after exposure with a LOEL of 99 mg/kg 2,4DNT and LOEL of TNT at 192 mg/kg. Changes in total protein paralleled those of albumin (data not shown). Glucose was elevated at 48 h with 2,4DNT (high dose) and reduced at 48 h with 4ADNT (LOEL 47 mg/kg). Serum sodium levels were decreased at 24 h after 2,6DNT (199 mg/kg) and 2ADNT (348 mg/kg) exposures and at 48 h after 2,4DNT (LOEL 198 mg/kg) and TNT (384 mg/kg) exposures. No significant treatment-related changes were observed for total bilirubin, creatinine or urea (data not shown).

Serum alanine aminotransferase (ALT) activity exhibited a dose-dependent decrease to 33% of vehicle control at 24 h after 4ADNT exposure with doses greater than 5 mg/kg. This effect was not sustained to 48 h post-exposure, but rather an increase was observed for the 94 mg/kg group. A similar trend of lesser magnitude occurred with serum aspartate aminotransferase (AST). Small declines in serum transaminase activities are occasionally observed with some chemicals often due to inhibition of enzymatic utilization of cofactor pyridoxyl-5′-phosphate; however, this effect is thought to be of relatively minor toxicological significance [17].

**Figure 1:**
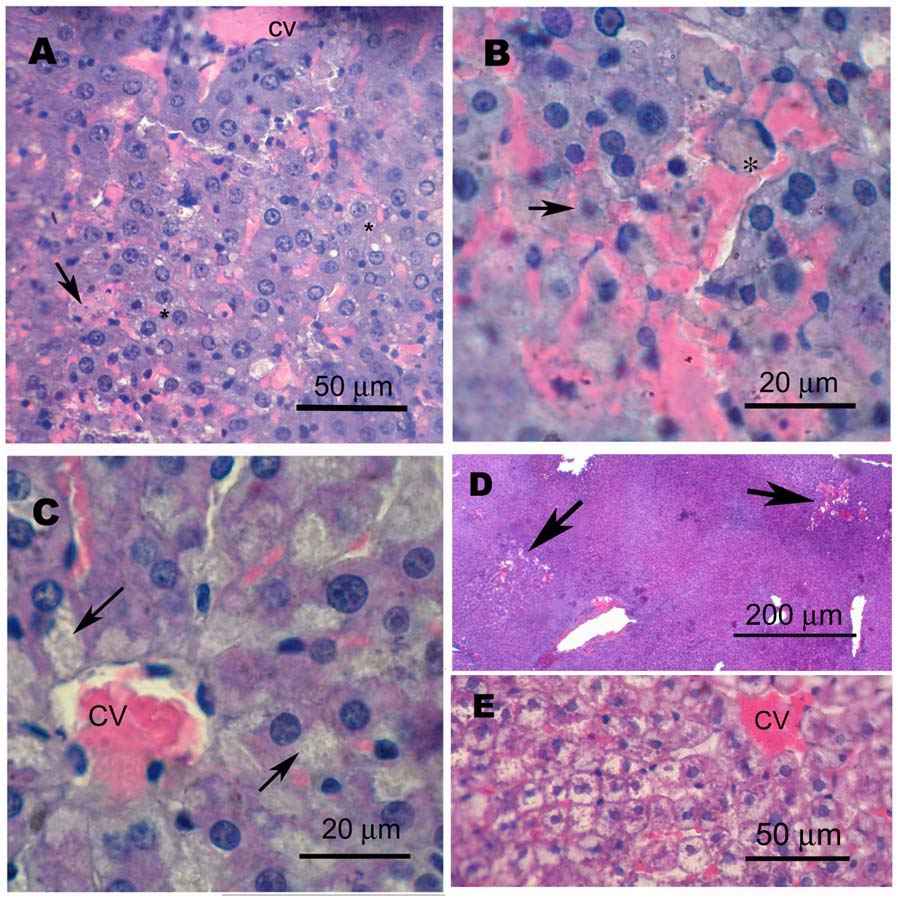
Pathology of livers from rats treated 48 h earlier with 50 mg/kg 2,6DNT (A,B) or 384 mg/kg TNT (C). Photomicrographs are from 5 µm sections stained with hematoxylin and eosin. Livers from one of 5 rats treated with 50 mg/kg 2,6DNT exhibited sloughing of hepatocyte remnants into congested sinusoids (A, arrow) and microvesiculated hepatocytes (A, *). A necrotic hepatocyte with pyknotic nucleus displaced from the liver cord by infiltrating erythrocytes is shown in B (arrow). Numerous apoptotic cells (B, *) were also seen. Amorphous vacuoles occupying a considerable portion of hepatocyte cytoplasms were numerous in livers of 3 of 5 rats treated with 384 mg/kg TNT (C, arrows). One rat treated with 94 mg/kg 4ADNT had focal hemorrhagic areas in the liver (D, arrows), while livers of other rats treated with 94 and 197 mg/kg 4ADNT exhibited ballooning degeneration (E).

One rat in each of the 50 and 99 mg/kg 2,6DNT groups (48 h) had serum ALT values in excess of 3000 IU/ml that thus caused their treatment means to exceed 7 times the control mean with ∼200% coefficient of variation (Table 1). Histopathology verified that these values truly reflected hepatotoxicity of sensitive rats within their treatment groups (Figure 1A and 1B). Livers of these rats contained congested sinusoids, some with sloughed hepatocyte remnants (arrow, Figure 1A). Segmented neutrophils were frequent, but confined to the sinusoids. Central veins and lining endothelia appeared normal with unaffected proximal hepatocytes in radiating cords. However, several cords become disorganized at midzonal regions where erythrocytes appeared to infiltrate and adjacent hepatocytes exhibited pyknotic nuclei (arrowhead, Figure 1B) and microvesiculated cytoplasm (asterisks, Figure 1A). In addition, apoptotic hepatocytes were evident (asterisk, Figure 1B). Others have shown necrosis and hemorrhage in livers of rats with 50% lethality at 24 h after 50 mg/kg 2,6DNT, i.p., while equivalent treatment with 2,4DNT was without effect [8].

**Figure 2 pone-0014662-g002:**
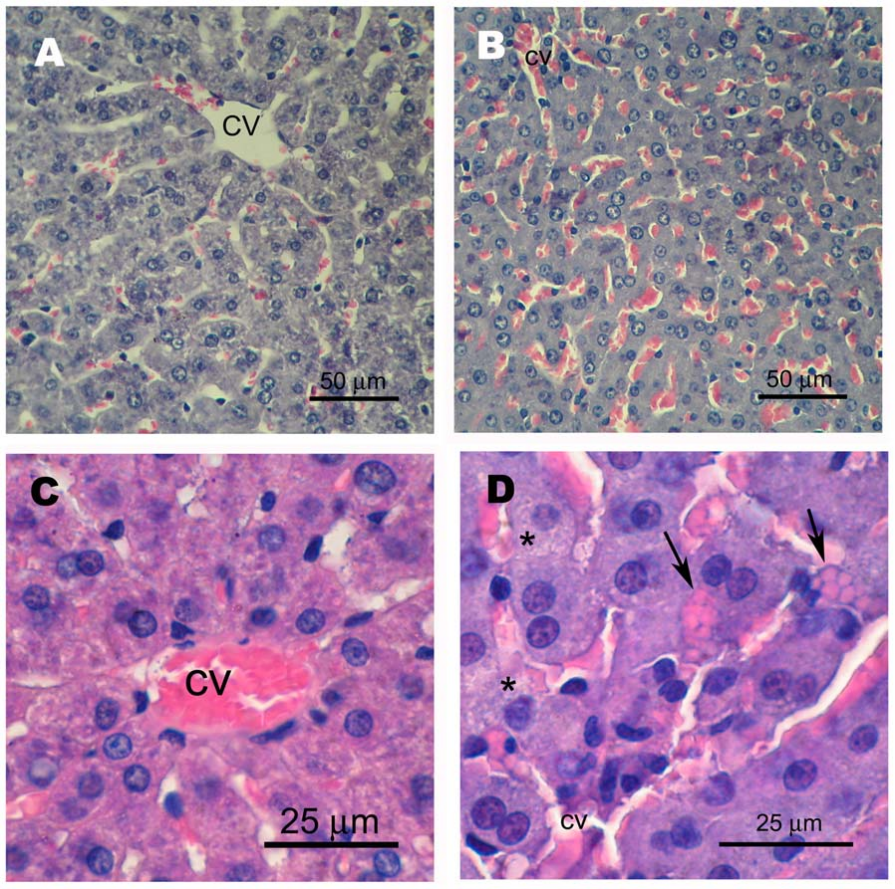
Representative photomicrographs of livers from rats treated 48 h earlier with vehicle (5% DMSO in corn oil, A and C) or 199 mg/kg 2,6DNT (B and D). Photomicrographs are from 5 µm sections stained with hematoxylin and eosin. Extensive sinusoidal congestion of 2,6DNT-treated rats is shown in panel B and compared to unaffected liver of vehicle treated controls in panel A. Higher power magnification illustrates infiltration of hepatocyte cords by erythrocytes (D, arrows) and microvesiculation (D, *) seen in livers of 2,6DNT-treated rats. Unaffected liver of vehicle-treated controls at high magnification is shown in panel C.

In contrast, hepatocytes of rats treated with the higher dose of 2,6DNT whose serum ALT levels were equivalent to vehicle controls appeared mostly undamaged (Figure 2B), however, sinusoids were markedly congested. Occasional erythrocyte infiltration between adjacent hepatocytes within a cord was noted (arrow, Figure 2D), but without associated necrosis as seen in the sensitive animals (arrowhead, Figure 1B). Enlarged nucleoli were evident in several hepatocytes of treated rat liver in agreement with ultrastructural changes after single dose dinitrotoluene reported previously [18].

Sinusoidal congestion was also evident with high dose 2,4DNT. Cytoplasmic amorphous inclusions were noted in hepatocytes of 3 livers and microvesicles in another of the 5 rats treated with 384 mg/kg TNT (Figure 1C). Livers of 4 of 5 rats treated with high dose TNT had regions where sinusoids were congested, but less area was involved than seen with dinitrotoluene-treated rats. Focal hemorrhages were seen in the liver of one rat at 48 h after treatment with 94 mg/kg 4ADNT (Figure 1D), while ballooning degeneration was common in livers from other rats 48 h after 94 and 187 mg/kg 4ADNT (Figure 1E). No pathological lesions were seen in livers of rats treated with 2ADNT.

Relative liver weights of rats treated with 4ADNT increased from 4.12±0.11% to 4.82±0.05%, 4.77±0.05% and 4.76±0.18% (means±SEM, n = 5) at 48 h after treatment with 93, 187 and 374 mg/kg, respectively. TNT at 384 mg/kg was associated with an increased liver weight of 4.90±0.23% relative to 4.03±0.17% for controls. The 99 mg/kg 2,4DNT group also had an elevated relative liver weight of 4.59±0.09% compared to concurrent vehicle control (3.85±0.08%), but higher doses (199 mg/kg = 4.06±0.25; 389 mg/kg = 4.29±0.18) were unaffected. Liver weights of rats treated with 2,6DNT and 2ADNT were unchanged from vehicle controls at 48 h post-treatment.

**Figure 3 pone-0014662-g003:**
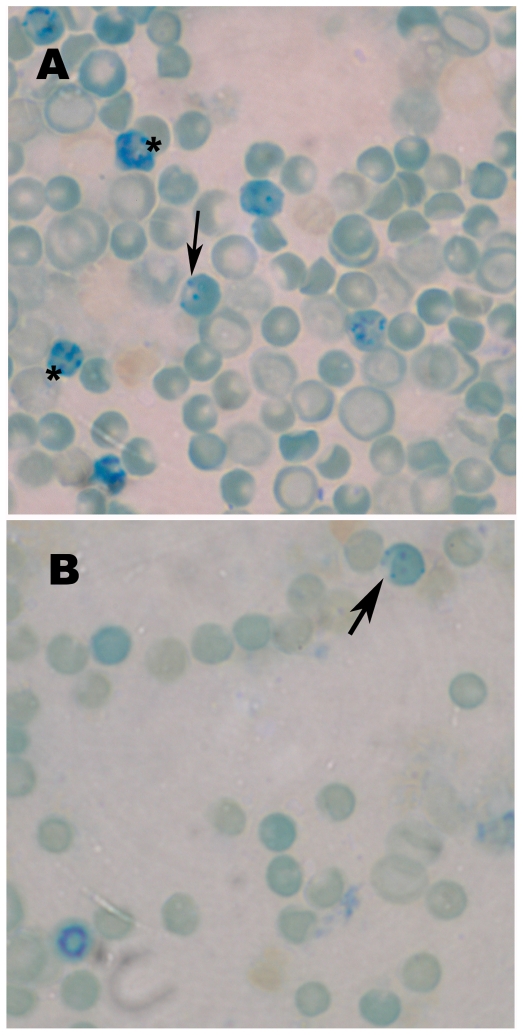
Morphology of erythrocytes from rats treated 24 h earlier with 2,6DNT. Photomicrographs at high magnification (100X objective with oil) of wedge preparations of blood vitally stained with new methylene blue are shown. Blood from rats treated with 199 (A) and 99 (B) mg/kg 2,6DNT are shown. Reticulocytes with blue stained mRNA are indicated by asterisks and arrows point to erythrocytes with indentations (bite cells).

The most notable finding from the hematology panel was an elevation of several related parameters indicating erythrocytosis associated with the dinitrotoluenes (Tables 3, 4). Erythrocyte count was elevated at 99 mg/kg at 24 h post-exposure and at 199 mg/kg and above at 48 h for both compounds. Increases in hemoglobin concentration and hematocrit paralleled that of erythrocyte count. For all parameters, the magnitude of change was greater for 2,6DNT than 2,4DNT. At 48 h after 199 mg/kg 2,6DNT, hemoglobin and hematocrit reached high values of 20 g/dL and 61% respectively. 2,6DNT, but not 2,4DNT, produced an increase in blood reticulocytes and was associated with mature erythrocytes with Heinz bodies and “bite cells” (Figure 3). TNT-treated rats exhibited increases in erythrocyte count and hemoglobin 24 h post-exposure at 192 and 384 mg/kg, respectively, but these effects were not sustained to 48 h nor of the magnitude seen with 2,6DNT. In contrast, blood erythrocyte parameters showed downward trends with 4ADNT as evidenced by ∼10% declines in hemoglobin and hematocrit at 48 h after treatment with 94 mg/kg and above (Tables 3, 4). Treatment with 2ADNT was without effect.

**Table 3:** Hematology Outcomes Affected by 2,4- and 2,6-DNT and TNT^a^.

Dose	Time	Granulocytes	Lymphocytes	Erythrocytes	HbG^b^	HCt	RDW	Retic
mg/kg	hr	10^6^/mL	10^6^/mL	10^9^/mL	g/dL	%	%	%
				**2,4DNT**				
0	24	0.23±0.15	2.94±0.63	7.20±0.14	13.7±0.2	41.3±0.8	10.9±0.1	3.0±0.1
	48	0.52±0.13	4.21±0.36	6.65±0.09	13.0±0.1	40.3±0.3	11.2±0.1	
5	24	0.52±0.14	3.27±0.69	7.37±0.18	13.9±0.2	42.2±0.5	11.0±0.1	2.5±0.1
	48	0.77±0.25	3.11±0.66	6.83±0.07	12.8±0.3	40.1±0.3	10.7±0.3	
50	24	0.51±0.21	3.12±0.46	7.63±0.11	14.3±0.3	44.0±0.6*	11.0±0.1	2.0±0.2
	48	0.98±0.20	4.31±1.22	6.93±0.08	13.3±0.1	41.6±0.4	11.1±0.1	
99	24	1.50±0.15**	4.84±0.60	7.96±0.08**	14.3±0.2	44.6±0.5**	11.2±0.2	2.3±0.2
	48	1.06±0.15	2.54±0.71	6.63±0.06	12.4±0.3	39.8±0.5	10.9±0.1	
198	24	1.02±0.25*	3.09±0.64	7.65±0.04*	14.2±0.1	42.9±0.4	10.9±0.1	2.5±0.4
	48	4.73±0.60	3.01±0.22	8.96±0.43**	17.0±0.9**	53.6±2.0**	11.5±0.1	
398	24	3.6, 2.4	1.8, 3.5	8.53, 6.04	15.3, 12.7	45.0, 39.3	10.6, 10.6	3.3, 3.3
	48	8.38±2.94**	0.97±0.23*	8.69±0.16**	16.5±0.2**	51.8±0.9**	12.0±0.7	
				**2,6DNT**				
0	24	0.36±0.22	6.16±1.23	7.39±0.18	13.9±0.3	43.4±0.9	11.3±0.1	3.4±0.2
	48	0.68±0.18	3.86±0.30	7.11±0.11	14.1±0.1	43.1±0.3	11.2±0.1	
5	24	0.29±0.13	5.12±0.85	7.57±0.20	14.1±0.3	44.8±1.1	11.0±0.2	3.0±0.2
	48	0.58±0.21	3.50±0.36	7.32±0.13	14.1±0.2	43.3±0.7	11.3±0.2	
25	24	0.20±0.08	5.42±0.82	7.94±0.29	15.1±0.6	46.7±1.8*	11.0±0.1	3.1±0.2
	48	0.49±0.09	3.56±0.52	6.96±0.12	13.6±0.1	41.2±0.4	10.8±0.1	
50	24	0.76±0.08	5.25±0.90	8.03±0.18	14.6±0.2	46.1±1.1	11.1±0.1	3.7±0.1
	48	1.45±0.35	4.95±0.25	7.47±0.26	14.4±0.5	43.6±1.5	11.1±0.2	
99	24	0.83±0.14	3.96±0.37	8.43±0.17**	15.3±0.3*	48.8±1.1*	11.0±0.1	4.1±0.2*
	48	1.36±0.66	4.86±0.50	7.58±0.21	14.7±0.4	44.6±1.2	11.1±0.1	
199	24	0.78±0.22	1.52±0.39**	8.02±0.12	14.6±0.2	45.7±0.8	11.0±0.2	4.9±0.1**
	48	7.58±0.99**	5.46±2.05	11.43±0.94**	20.4±1.3**	60.6±4.9**	12.5±0.4**	
				**TNT**				
0	24	0.33±0.17	6.22±0.79	7.62±0.14	14.5±0.2	44.9±0.8	10.9±0.2	
	48	1.06±0.18	4.09±0.26	7.36±0.38	13.6±0.3	42.6±1.2	10.9±0.4	
5	24	0.43±0.11	5.33±0.73	7.08±0.23	13.6±0.3	41.6±1.4	10.6±0.2	
	48	0.97±0.23	4.55±0.46	7.70±0.41	13.8±0.3	45.0±2.7	11.1±0.3	
48	24	0.23±0.09	5.93±0.82	7.85±0.25	14.4±0.3	46.3±1.4	10.8±0.2	
	48	1.00±0.26	4.79±0.92	7.12±0.07	13.7±0.1	41.6±0.2	10.6±0.1	
96	24	0.60±0.49	4.76±0.37	7.79±0.11	14.7±0.2	45.6±0.7	11.0±0.2	
	48	0.99±0.19	4.95±0.36	7.53±0.51	13.7±0.4	43.1±3.0	11.2±0.2	
192	24	2.16±1.02	2.95±0.46**	8.61±0.41*	15.1±0.3	48.8±2.3	10.9±0.2	
	48	1.42±0.21	4.01±0.46	7.99±0.63	13.1±0.4	46.3±3.6	11.5±0.2	
384	24	4.26±2.03*	1.89±0.30**	8.31±0.19	16.1±0.2**	45.2±0.5	10.7±0.1	
	48	2.60±0.71*	4.28±0.87	7.36±0.38	12.8±0.3	40.6±1.3	12.7±0.2**	

a Values are means±SEM for n = 5, except for 199 and 398 mg/kg 2,4DNT at 48 h where n = 4 and 199 mg/kg 2,6DNT at 48 h where n = 3 and for 398 mg/kg 2,4DNT at 24 h where individual values are shown. Means that differed from concurrent vehicle controls (0 mg/kg) are indicated by * or ** for p<0.05 or 0.01, respectively.

b Abbreviations. HbG = hemoglobin, HCt = hematocrit, RDW = red blood cell distribution width, Retic = reticulocytes.

**Table 4:** Effect of 2ADNT and 4ADNT on Hematology Outcomes^a^.

Dose	Time	Granulocytes	Lymphocytes	Erythrocytes	HbG^b^	HCt	RDW
mg/kg	hr	10^6^/mL	10^6^/mL	10^9^/mL	g/dL	%	%
				**2ADNT**			
0	24	0.54±0.19	5.07±0.56	7.70±0.15	14.7±0.3	43.6±0.7	10.6±0.1
	48	0.76±0.19	3.75±0.50	6.83±0.07	13.2±0.1	41.2±0.3	11.1±0.0
4	24	0.85±0.06	5.75±0.40	7.67±0.07	14.7±0.1	44.0±0.4	10.6±0.1
	48	0.40±0.15	3.71±0.56	6.80±0.10	13.1±0.2	40.7±0.6	11.2±0.1
44	24	0.60±0.19	4.23±0.21	8.05±0.11	14.5±0.6	45.8±0.4	10.8±0.1
	48	0.45±0.14	4.13±0.36	7.05±0.16	13.6±0.2	42.4±0.8	11.0±0.1
87	24	0.73±0.25	3.46±0.37	7.41±0.27	14.3±0.4	42.9±1.7	10.6±0.1
	48	0.83±0.11	4.01±0.24	6.95±0.14	13.4±0.3	41.7±0.9	10.9±0.1
174	24	0.70±0.17	3.94±0.40	7.37±0.19	14.7±0.3	42.7±1.1	10.6±0.1
	48	0.92±0.21	4.62±0.972	7.05±0.19	13.3±0.4	41.5±1.1	10.9±0.1
348	24	0.50±0.18	3.84±0.14	7.66±0.14	15.3, 12.7	43.9±0.9	11.0±0.1
	48	0.76±0.10	3.40±0.36	6.80±0.08	12.7±0.1	40.3±0.5	10.9±0.2
				**4ADNT**			
0	24	1.22±0.17	5.26±0.60	7.44±0.20	14.5±0.3	42.8±1.0	11.0±0.1
	48	1.09±0.369	4.80±0.81	7.14±0.12	13.4±0.3	41.7±0.6	10.8±0.1
5	24	0.88±0.13	4.30±0.59	7.77±0.11	14.9±0.3	44.6±0.7	10.6±0.1
	48	1.92±0.40	5.48±0.62	6.67±0.11	12.8±0.2	39.4±0.5	10.4±0.1
47	24	1.20±0.09	4.56±0.31	7.82±0.13	15.1±0.1	45.0±0.4	10.9±0.1
	48	1.87±0.24	5.52±0.90	6.94±0.12	13.3±0.1	40.9±0.4	10.5±0.1
94	24	1.08±0.15	4.30±0.32	7.60±0.14	14.7±0.4	43.6±1.0	10.7±0.1
	48	1.77±0.53	5.74±1.10	6.45±0.17*	12.2±0.2**	37.8±0.7**	10.8±0.1
187	24	1.08±0.04	4.71±0.32	7.66±0.05	14.9±0.2	44.6±0.3	10.7±0.1
	48	1.06±0.29	4.59±0.36	6.63±0.16	12.5±0.3*	39.0±0.8*	10.6±0.1
374	24	1.30±0.16	3.78±0.30	7.76±0.19	15.1±0.3	44.4±0.9	10.7±0.1
	48	2.15±0.37	4.97±0.78	6.92±0.14	13.1±0.2	41.0±0.7	10.6±0.2

a Values are means±SEM for n = 5. Means that differed from concurrent vehicle controls (0 mg/kg) are indicated by * or ** for p<0.05 or 0.01, respectively.

b Abbreviations. HbG = hemoglobin, HCt = hematocrit, RDW = red blood cell distribution width.

Marked elevation of blood granulocytes were noted at 48 h after high dose 2,4DNT and TNT and 199 mg/kg 2,6DNT (3). Earlier elevations occurred with 2,4DNT at doses of 99 mg/kg and above and with 384 mg/kg TNT. Granulocytosis at 24 h for TNT and at 48 h for 2,4DNT tended to parallel a decrease in lymphocytes as often occurs with corticosteroid-mediated stress [17]. In contrast, leukocyte counts of rats treated with the amino-dinitrotoluenes were unaffected by treatment at either time point (Table 4).

### Dose responsive differentially expressed gene numbers in liver affected by nitrotoluenes

**Figure 4 pone-0014662-g004:**
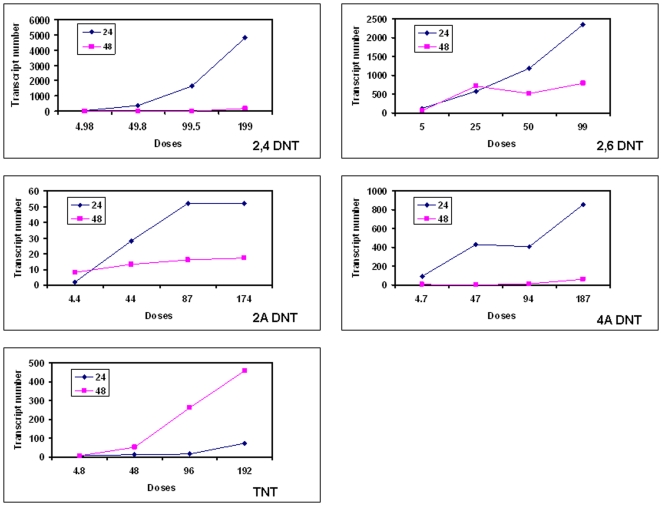
The dose and time dependent numbers of transcripts regulated by 2,4DNT, 2,6DNT, 2ADNT, 4ADNT or TNT. Rats were exposed to one of the 5 compounds for 24 h (blue line) or 48 h (red line), with one of 4 doses plus vehicle controls. Rats were sacrificed and liver tissues were employed for total RNA isolation and microarray hybridization and analysis as described in Materials and Methods. The numbers of transcripts at Y axis were achieved by comparing the gene expression profiles of animals treated with one of four doses with respective control animals at the time point 24 h or 48 h for one of the compounds.

A strong dose responsive change in the total number of differentially expressed genes was observed at both time points for all compounds (Figure 4). The dose responsive change suggests that the doses are in an appropriate linear range for the microarray experiment. The number of statistically regulated transcripts was consistently larger at 24 h than 48 h for all compounds except TNT. TNT was reversed in comparison to the other 4 compounds in that it produced a higher number of altered transcripts at 48 h. In light of the chemistry data, metabolism of TNT into 2,6DNT and 2,4DNT in liver may cause a lag in a decrease of observed impacts upon gene expression.

**Figure 5:**
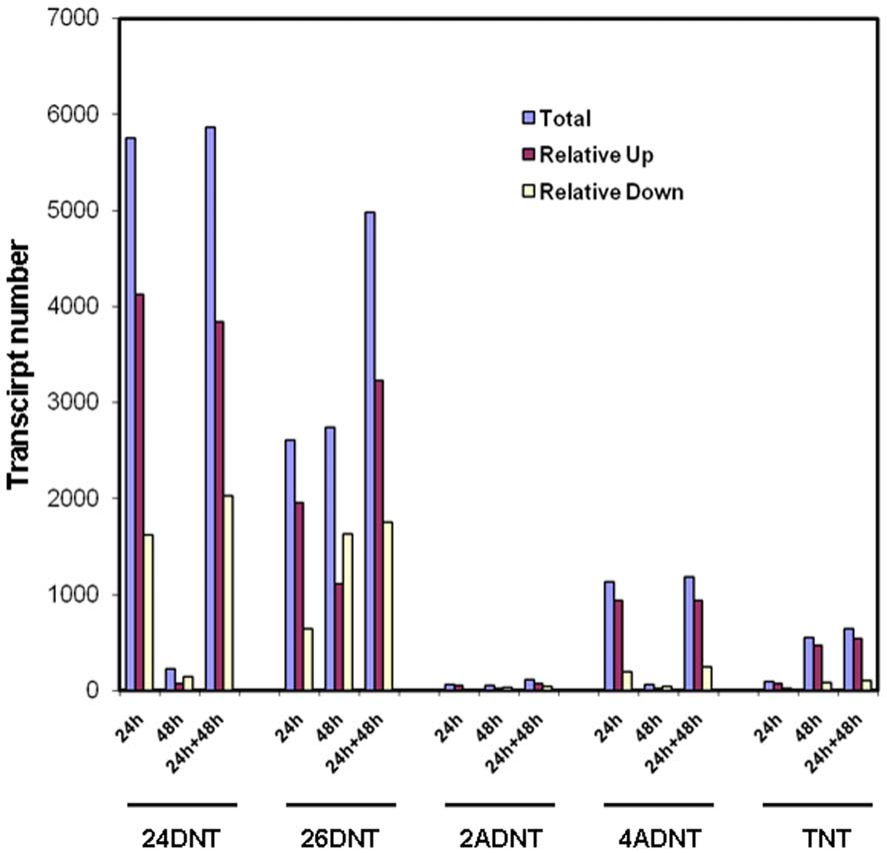
Total transcript numbers regulated by the five compounds. Rats were treated with different compounds with variant doses and microarray experiments were performed as described in Materials and Methods. The number of transcripts that were at each time point was analyzed using One-Way ANOVA with a Benjamin-Hochberg false discovery rate less than 0.05 across various doses at each time point (24 h or 48 h) for each compound. The total regulated transcript number for each compound was calculated by summarizing the numbers of both time points (24 h+48 h), and overlapped transcripts were only counted once. The relative up or down-regulated transcripts were obtained by comparing averaged animal samples with various dose treatments with respective control animal samples at each time point or both time points.

While the dose responsive pattern of the number of gene expression was similar for the compounds examined (Figure 4), there was a large difference in the total number affected by the chemicals (Figure 5). We observed a remarkable difference in the number of altered genes for both time points together (24 h+48 h) induced by the 5 compounds (Figure 5). 2,4 DNT displayed the highest changed number (close to 6000) at 24 h+48 h, followed by 2,6 DNT (nearly 5000), 4ADNT (over 1000), TNT (nearly 700) and 2ADNT (110) (Tables S1, S2, S3, S4, S5, S6, S7, S8, S9, S10). The identification of very few differentially expressed transcripts by 2ADNT relative to the other nitrotoluenes is consistent with observed clinical effects suggesting that reduction of the nitroso group to an amino group at ring position C-2 dramatically reduces the biological effects of nitrotoluenes.

Next, we determined the amount of up-regulated or down-regulated transcript numbers for the compounds. To do that, we compared treated and control samples at 24 h or 48 h or both time points together for each compound. Based on significantly regulated transcripts, if the averaged normalized intensity of a gene was higher in the treated group than the control group, we considered the gene relatively up-regulated; and if lower, down-regulated. According to this criterion, we found another common phenomenon: there were much more up-regulated transcripts than down-regulated transcripts at 24h and both time points together (24 h+48 h) (Figure 5) for 2,4DNT, 2,6DNT, 2ADNT, 4ADNT and TNT. Interestingly, at 48 h, there were far more down-regulated transcripts than up-regulated transcripts for the 4 compounds 2,4DNT, 2,6DNT, 2ADNT, 4ADNT. TNT showed a reverse trend once again in that it induced more up-regulated transcripts at 48 h than down-regulated ones. Our results indicate that not only was a trend conserved for the dose responsive effects, but also a time –responsive up and down-regulated trend among these compounds was conserved, implicating that a common underlying mechanism may be shared by these compounds.

### Comparison of general gene expression profiles induced by the 5 compounds

**Figure 6:**
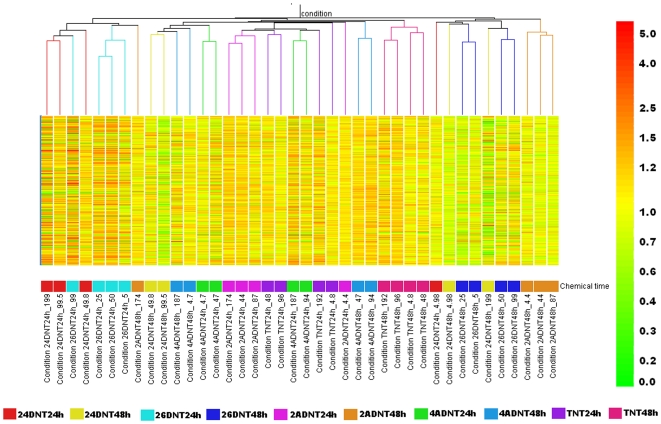
Hierarchical clustering of experimental conditions. Experimental conditions were based on averaging samples with the same dose treatments of a compound for 24 h or 48 h and total 40 experimental conditions were used for the clustering. Transcripts used for making these conditional trees were selected based on filtering on flags. Only a transcript that was presented in at least 50% samples was chosen for clustering. Pearson correlation algorithm with average linkage was conducted to calculate distances between conditions.

The overall gene expression profiles induced by the 5 chemicals with various doses and different times were compared by two-way hierarchical clustering of averages of replicates within each of the 40 conditions (Figure 6). The dendrogram was clearly divided into 3 groups. Eight conditions formed the first group containing 3 conditions from 2,4DNT, all 4 conditions from 2,6DNT at 24 h, and one from 2ADNT at 48 h. The second group included 22 conditions. Interestingly, this group was almost entirely composed of conditions from 2ADNT, 4ADNT and TNT except two conditions from 2,4DNT, 48 h. The third group contained 10 conditions in which two distinctive subgroups were formed. One subgroup contained 3 conditions that were from 2ADNT at 48 h, while the other subgroups included conditions from 2,4DNT at 48 h and 2,6DNT at 48 h. Experimental conditions with the same chemical dose and time were likely to cluster together. Conditions within the same dose and the same compound from different time points (24 h or 48 h) seldom clustered together in a subgroup indicating that the time effect is much stronger than the dose effect for all the compounds.

From Figure 6, most of the gene expression profiles of 2,4DNT and 2,6DNT clustered together and most of the 2ADNT, 4ADNT and TNT induced gene expression profiles grouped together. From supplementary Figure 1A (File S1), we noticed that gene expression profiles of 2,4DNT and 2,6DNT clustered together and 2ADNT, 4ADNT and TNT induced gene expression profiles grouped together. We further made averaged conditions based on compound treatments and ignored dose and time effects. A simple condition tree using the same flagged transcripts is shown in supplementary Figure 1B. 2,4DNT and 2,6DNT formed in one separate group, 2ADNT, 4ADNT and TNT were located in the other group in which 4ADNT and TNT were clustered together. Since 2ADNT and 4ADNT are metabolites of TNT, it is easy to understand that the 3 compounds form in a group. The expected results provide proof that this high-throughput experiment is reliable.

### Common gene expression patterns of significantly differentially expressed genes due to nitrotoluene exposure

**Figure 7:**
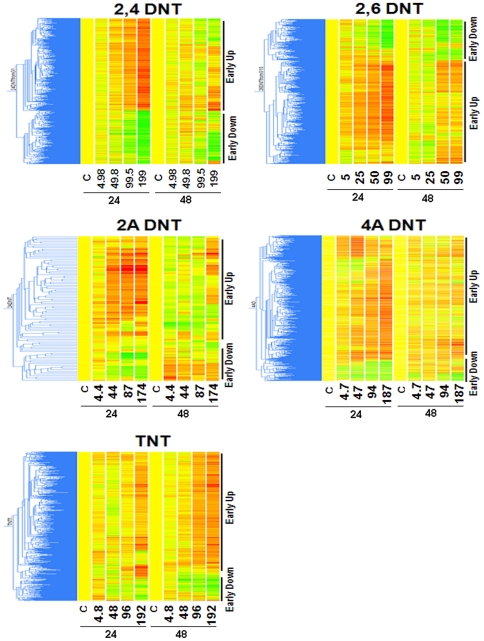
Hierarchical clustering of significantly regulated genes. Most significantly regulated transcripts of each compound were used for constructing gene clustering trees across different conditions including control and 4 different doses at 24 h or 48 h for each compound. Pearson correlation algorithm with average linkage was performed to calculate distances between transcripts. Two distinctive gene expression patterns: early up-regulated (early up) and early down-regulated (early down) gene expression patterns were indicated on the right of each cluster.

We also performed a hierarchical clustering per treatment. Because the two very strong gene regulators, 2,4DNT and 2,6DNT have over 2000 genes that were affected, in order to identify most significantly regulated genes, a more stringent false discovery rate (FDR) with adjusted p-value less than 0.002 was applied resulting in 1418 and 1241 significantly differentially expressed transcripts for 2,4DNT and 2,6DNT, respectively. For the other three compounds, no more stringent FDR was applied because their changed gene numbers were much less than 2000. One-way hierarchical clustering was performed across various doses and times per chemical. Interestingly, the 5 compounds share similar gene expression patterns (Figure 7). The genes were primarily divided into two distinctive expression patterns: early up-regulated (24 h) and early down-regulated (24 h). Other common patterns shared by the 5 compounds include a clear dose response for both up-regulated and down-regulated transcripts at 24 h. We also noticed that there were more up-regulated than down-regulated genes at 24 h for all the compounds which is consistent with Figure 5. For all the compounds but 2ADNT, transcripts generally up-regulated at 24 h were also up-regulated at 48 h as well. The common gene expression patterns shared by the compounds imply that these compounds may share some common mechanisms.

### Overlapping functional terms of differential genes among the 5 compounds

To gain insight into the functional categories of these significantly changed genes, we performed gene ontology (GO) analyses based on biological processes. Early up-regulated and early down-regulated gene groups for each compound across all doses (Figure 7) underwent GO analyses separately. We first examined the similarity of functional terms from the up-regulated gene groups between compounds (Table 5). The greatest overlap occurred between 2,4DNT and 2,6DNT. 4ADNT also had a relatively high similarity of functional terms with 2,4DNT and 2,6DNT (Table 5). These results are consistent with the comparison of general gene expression profiles (Figure 6 and File S1). Consistent with observations of little effect on biological parameters and differentially expressed genes, 2ADNT had very few significantly affected GO terms. Four significantly up-regulated functional terms were shared by all 5 compounds (Table 6). The top significantly changed GO terms regulated by all 5 compounds were “metabolic process” and “cellular metabolic process” (a child term under metabolic process), protein folding, and “xenobiotic metabolic process” (a child term of “response to xenobiotic stimulus”).

**Table 5:** Comparison of significantly functional terms up-regulated by the 5 compounds.

	2,4DNTup	2,6DNTup	2ADNTup	4ADNTup	TNTup
2,4DNTup	148(100%)	90(60.8%)	7(8.5%)	80(53.1%)	44(21.8%)
2,6DNTup		148(100%)	6(7.3%)	76(50.5%)	28(25.9%)
2ADNTup			15(100%)	14(16.6%)	10(24.1%)
4ADNTup				153(100%)	53(50.0%)
TNTup					68(100%)

Note: The numbers outside of a bracket are calculated based on the common functional terms between two compounds. The number inside a bracket is the similarity rate which is calculated as that the number of common functional terms divided by the averaged total functional terms of two compared compounds.

**Table 6:** Commonly most significantly up-regulated functional terms based on biological process of Gene Ontology in response to the compounds.

Functional terms	2,4DNT	2,6DNT	2ADNT	4ADNT	TNT
metabolic process	5.86E-14	4.63E-14	0.0062795	6.63E-20	1.09E-14
cellular metabolic process	3.86E-15	7.60E-14	0.007577	1.46E-22	8.44E-13
cellular macromolecule metabolic process	0.0072135	2.89E-07		4.56E-06	0.001380
cellular biosynthetic process	0.0046218	0.02843983		2.71E-12	1.64E-13
macromolecule metabolic process	9.03E-13	1.37E-15		4.79E-08	0.039807
cellular macromolecule metabolic process	0.0072135	2.89E-07		4.56E-06	0.001380
cellular protein metabolic process	0.0058248	2.67E-07		3.27E-06	0.002308
protein metabolic process	0.0038298	4.97E-09		7.26E-06	0.003270
cellular protein metabolic process	0.0058248	2.67E-07		3.27E-06	0.002308
protein folding	1.10E-05	1.99E-04	0.083242	3.49E-06	0.005908
RNA metabolic process	1.47E-11	3.04E-05		3.33E-04	0.004
primary metabolic process	1.13E-14	5.91E-14		2.76E-17	1.54E-08
protein metabolic process	0.0038298	4.97E-09		7.26E-06	0.003270
response to xenobiotic stimulus	0.0680837	0.005775329	8.52E-07	1.96E-04	1.43E-04
xenobiotic metabolic process	0.0626892	0.004970058	7.40E-07	1.61E-04	1.21E-04
macromolecule localization	4.38E-06	2.06E-07		3.73E-04	0.043349
protein localization	1.79E-05	3.46E-06		6.88E-04	0.030145
establishment of protein localization	3.49E-06	1.13E-06		0.001733	0.016687
protein transport	7.66E-06	2.73E-07		0.0010188	0.007891
Cellular process					
organelle organization and biogenesis	4.69E-06	8.47E-08		9.23E-06	0.049135
mitochondrion organization and biogenesis	0.0330679	7.40E-04		0.0066755	0.009797
intracellular transport	8.49E-07	3.94E-12		0.0032581	0.004579
intracellular protein transport	3.58E-07	1.46E-05		0.002939	0.008296
protein targeting	4.59E-07	3.97E-04		2.44E-04	0.013345

Twenty significantly up-regulated GO functional terms were shared by 2,4DNT, 2,6DNT, 4ADNT and TNT, among which at least half were child terms of “metabolic process” and “cellular metabolic process”. Primarily, these terms could be categorized as “macromolecule metabolic process”, “RNA metabolic process” and “cellular biosynthetic process”. The major term under macromolecule metabolic process was cellular protein metabolic process, under which a significant common term was protein folding.

The functional term “cellular biosynthetic process” was more enriched in response to TNT, 4A DNT than 2,4DNT and 2,6DNT treatments (Table 6). NQO1, represented in “xenobiotic metabolic process” and in the “cellular biosynthetic process”, was up-regulated by all 5 compounds indicating this gene may play an important role in the response to all 5 compounds.

Under the functional term “RNA metabolic process”, pirin (PIR) was up-regulated by 2,6DNT, 2ADNT, 4ADNT and TNT. Tryptophanyl-tRNA synthetase (WARSs) was induced by 2,6DNT, 4ADNT and TNT; small nuclear ribonucleoprotien polypeptide A (SNRPA), NHP2-like protien1 (NHP2L1) was up-regulated by 2,4DNT, 4ADNT and TNT. 2,4DNT, 2,6DNT and 4ADNT shared more induced genes of “RNA metabolic process” which included the gene for absent, small, or homeotic-like gene (ASH2), general transcription factor II H, polypeptide 1 (GTF2H1), EBNA1 binding protein 2 (EBNA1BP2), nucleolar protein 5A (NOL5A), peptidylprolyl isomerase (cyclophilin)-like 3 (PPIL3) and tRNA splicing endonuclease 2 homolog (*Saccharomyces*. *cerevisiae*) (TSEN2).

Other shared up-regulated functional terms included “mitochondrion organization and biogenesis” as well as “protein transport” (Table 6). Interestingly, some genes present in earlier categories such as P53, HSPD1 also fit into both categories. The gene translocase of inner mitochondrial membrane 9 (TIMM9) found under both categories, was more strongly induced by 2,4DNT and 2,6DNT.

Further comparison of up-regulated GO functional terms of 2,4DNT, 2,6DNT and 4ADNT revealed a high number of shared terms, 65 terms. “DNA metabolic process,” a child term of “nucleobase, nucleoside, nucleotide and nucleic acid metabolic process” and “biopolymer metabolic process”, was a key term shared by the 3 compounds.

When down-regulated GO biological process terms were compared, 2,4DNT and 2,6DNT were more similar than other compounds (Table 7). This high similarity is consistent with the up-regulated GO biological process terms and gene expression profiles as described earlier. Such correlation suggests that these two compounds have highly similar molecular modes of action. “Lipid biosynthetic process”, which is highly related to “lipid metabolic processes”, was affected by all compounds (Table 8). Two critical downstream GO terms of “lipid metabolic process” were “fatty acid metabolic process” and “sterol metabolic process”. “Amino acid metabolic process” and/or its child terms, “organic acid metabolic process” and “carboxylic acid metabolic process”, were significantly down-regulated by 2,4DNT, 2,6DNT, 2ADNT and TNT.

**Table 7:** Comparison of significantly functional terms down-regulated by the 5 compounds.

	2,4DNTdown	2,6DNTdown	2ADNTdown	4ADNTdown	TNTdown
2,4DNTdown	127(100%)	61(54.2%)	8(11.3%)	2(2.3%)	9(12.3%)
2,6DNTdown		98(100%)	6(10.3%)	2(5.6%)	7(11.9%)
2ADNTdown			18(100%)	4(20.0%)	6(12.4%)
4ADNTdown				22(100%)	4(21.1%)
TNTdown					19(100%)

**Table 8:** Commonly most significantly down-regulated functional terms based on biology process of Gene Ontology in response to the compounds.

Functional terms	2,4DNT	2,6DNT	2ADNT	4ADNT	TNT
Lipid metabolic process	1.39E-14	6.79E-12			0.00198
Lipid biosynthetic process	1.46E-10	0.000702	0.0159	1.46E-22	0.0129
Immune system process				1.99E-06	8.89E-05
Immune response	0.06268	0.004970	7.40E-07	1.61E-04	1.21E-04

The second common significantly down-regulated GO biological process term was “immune system process” under which “immune response” was also affected. Both terms were more enriched in 4ADNT and TNT exposures than with exposure to the other 3 compounds(Table 9). Our results suggest that these chemicals affect immune response and chemokine signaling.

**Table 9:** Most significantly regulated genes involved in immune response for the 5 compounds.

Compounds	Most significantly regulated genes
2,4DNT	C4A, C5,C9,CXCL12,DPP4,EGR1,ITIH1, KDR, LTB, MASP1, RT1-S3
2,6DNT	C4A,C4BPA,C5,C6,CFH,CFI,CXCL12,DPP4,EGR1,FOXN1,H2-T18,ITIH1,LTB,MASP1
4ADNT	C1QG,CCL6,CCR5,CD83,CORO1A,ERAF,FCGR3A,GBP2,IL1B,IL6RA,LTB,LY86,PRKR,RT1-DA,RT1-S3,RT1-T24-1,SPN
TNT	CCL2,CXCL10,CXCL9,CXCL13, CXCL12, OASL2,RT1-BB,RT1-DA,RT1-S3,TNFRSF14

### Pathways influenced by all the 5 compounds

**Figure 8:**
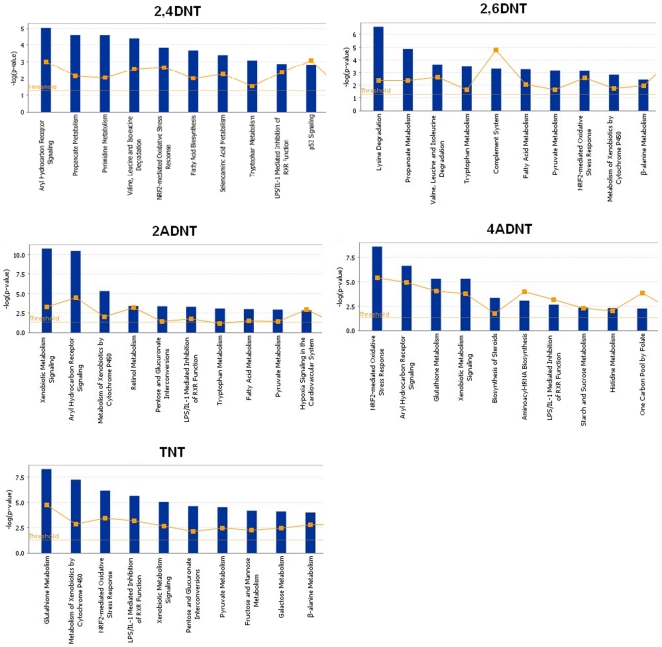
Most significantly canonical pathways regulated by the 5 compounds. Top ten pathways were selected to present for each compound. Most significantly regulated transcripts of each compound were chosen to run the Ingenuity pathway tool. The bigger the -log(p-value) of a pathway is, the more significantly the pathway is regulated. The threshold lines represent a p value with 0.05.

To further explore the molecular mechanisms, we used the most significantly differentially expressed transcripts of each chemical exposure to perform canonical pathway analyses. In general, a number of pathways were common between any compound pair (Table 10). Five pathways were strongly affected by all 5 compounds (File S1). The common pathways were also generally present in the top significant pathways affected by the compounds (Figure 8). The most significantly regulated genes are listed in File S1 for each pathway regulated by each compound. There were 5 pathways that were significantly regulated by 4 of the 5 compounds (File S1). Xenobiotic metabolism signaling and metabolism of xenobiotics by cytochrome P450 pathways were highly enriched in 2,6DNT, 4ADNT, 4ADNT and TNT exposure gene expression profiles. Propanoate metabolism, fatty acid metabolism and valine, leucine and isoleucine degradation pathways were strongly affected by 2,4DNT, 2,6DNT, 2ADNT and TNT. Nearly half of these 10 commonly impacted pathways (File S1), belong to amino acid metabolism, suggesting that amino acid metabolism plays a critical role in the compounds' effects which is consistent with Gene Ontology analysis.

**Table 10:** Comparison of significantly canonical pathways regulated by the 5 compounds.

	2,4DNT	2,6DNT	2ADNT	4ADNT	TNT
2,4DNT	44(100%)	18(46.8%)	15(38.9%)	12(31.2%)	17(43.0%)
2,6DNT		30(100%)	15(47.6%)	10(31.7%)	17(52.3%)
2ADNT			33(100%)	14(42.4%)	23(67.6%)
4ADNT				33(100%)	16(47.1%)
TNT					35(100%)

Note: The number outside of a bracket is the common pathway number between two compounds. The number inside a bracket is the similarity rate which is calculated as that the number of common pathways divided by the total averaged pathway terms of two compared compounds.

Examination of the most significantly regulated genes in pathways reveals that a number of genes from a few different gene families play a repeated role in multiple pathways that were regulated by chemical exposures. These gene families included aldehyde dehydrogenase 1 (ALDH1), gluathione S-transferase (GST), heat shock protein (HSP), and cytochrome P450 (CYP) families. ALDH1 family is involved in all the common pathways except NRF2-mediated oxidative stress response. The most significant differentially expressed genes under this family included ALDH1A1, ALDH1A3, ALDH1B1, ALDH1L1, and ALDH1L2. They were strongly up-regulated by at least two compounds, with some members affected by 3 compounds. For example, the family member ALDH1B1 was regulated by 2,4DNT, 2ADNT, and 4ADNT while ALDH1L1 was significantly affected by 2,4DNT, 4ADNT and TNT. Several genes in the GST family including GSTM1 (muscle), GSTM2, GSTM3, GSTM4 or GSTM7-7, GSTM5, GST pi1(GSTP1) and GST theta 1(GSTT1) participate in at least half of the commonly affected pathways (File S1). These genes were more likely to be up-regulated by the compounds.

The most significantly regulated genes shown in the commonly affected pathways in the HSP family included HSP 90KDa alpha (cytosolic), class A member 1(HSP90AA1), heat shock 27kDa protein 1 (HSPB1), HSP 90kDa alpha (cytosolic), class B member1 (HSP90AB1), and heat shock 22kDa protein 8 (HSPB8). These genes were usually up-regulated and involved in the following common pathways: aryl hydrocarbon receptor signaling, xenobiotic metabolism signaling, and NRF2-mediated oxidative stress response. We have found that HSP genes were highly enriched in the GO biological process term, “response to xenobiotic stimulus”. Since xenobiotic metabolism signaling also encompasses aryl hydrocarbon receptor signaling, and NRF2-mediated oxidative stress response, the pathway analysis results are consistent with the GO analysis indicating that heat shock proteins play an important role in the response to exposure to the 5 compounds.

A large number of differentially expressed genes belonged to the cytochrome P450 family (CYP1A1, CYP1A2, CYP3A5, CYP3A43, CYP4A11, CYP7A1, CYP51A1, CYP2C7, CYP2C44, CYP2C70, CYP2D6, CYP2D12, CYP2D26, CYP4F8, and CYP2J9. They mainly took part in the following common pathways: aryl hydrocarbon receptor signaling, LPS/IL-1 mediated inhibition of RXR function, xenobiotic metabolism signaling and metabolism of xenobiotics by cytochrome P450. These CYP family genes were primarily up-regulated by all the compounds.

Although there was no common significant cell death signaling pathways involved in the expression profiles induced by these compounds, different pathways associated with cell death signaling were strongly impacted. Apoptosis and/or P53 signaling were evidently affected by two and more of these compounds. Three genes which were commonly regulated by two or more compounds were P53, Bcl2-associated X protein (Bax), and caspase 3(CASP3). Some pathways such as estrogen receptor pathway and nucleotide excision repair pathway were only significantly regulated by 2,4DNT and 2,6DNT. Antigen presentation pathway was only evidently affected by TNT.

### Nitrotoluenes impact common gene networks

**Figure 9:**
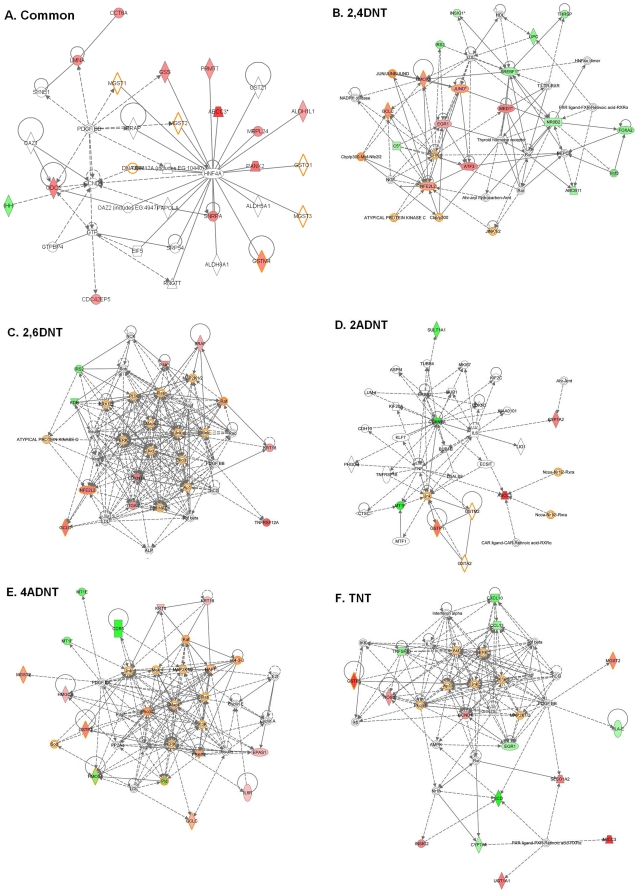
Common and specific gene networks related to liver toxicity induced by the compounds. The common gene network (A) was constructed using commonly significantly regulated genes by at least four of the compounds. For constructing specific gene networks related to liver toxicity of 2,4DNT(B), 2,6DNT(C), 2ADNT(D), 4ADNT(E) and TNT(F), only significantly regulated genes involved in known function to liver or hepatoxicity or liver diseases were selected for each compound. The red and green highlighted genes represent up-regulated and down-regulated genes respectively by one of the compound. The orange highlighted genes are involved in NRF2-mediated oxidative stress response. The up or down-regulated genes in the networks were determined by comparing the averaged treatments with relative controls of a compound. The gene networks were built using the Ingenuity knowledge base tool. The networks scores described in Materials and Methods for all the networks were bigger than 10.

To further understand the common mechanisms, we looked for differentially expressed genes in common with all five chemical exposures. Fifty-four transcripts corresponding to 47 non-redundant genes were significantly affected by at least four of the 5 compounds (Table S11). We constructed several networks using the 47 genes. Interestingly, one significant network was associated with hepatic system disease and liver cholestasis (Figure 9A) suggesting this network could present a common mechanism for possible induction of hepatoxicity seen with exposure to 2,4DNT, 2,6DNT and TNT. Although no adverse effects were seen with the chemicals 2ADNT and 4ADNT, the shared network suggests the potential for some effects similar to the other chemicals under prolonged exposure conditions. Several shared up-regulated genes including ABCC3, PRMT7, GSS, SNRPA, PANX2, MRPL24, and ALDH1L1 all connected with hepatocyte nuclear factor 4 alpha (HNF4A). While HNF4A was not a common altered gene, as a central transcription factor in this network, it may be important in modulating the effects caused by these chemicals. Genes involved in NRF2-mediated oxidative stress response pathway were also found in the network.

Based upon evidence that the nitrotoluenes impacted a common gene network related to liver effects, we next examined each chemical exposure for specific gene networks related to liver function. To fulfill this goal, we employed the significantly changed genes by each compound that have been known to be associated with liver function to build gene networks based on Ingenuity knowledge base. Specific significant gene networks that were associated with hepatic system function, liver cholestasis and/or liver damage were generated for each compound (Figures 9B–F). Several transcription factors such as NFE2L2 (NRF2), EGR1, JUND, and SREBF1 were highly connected in the 2,4DNT affected gene network, indicating these genes may play a role in 2,4DNT impacts on liver (Figure 9B). In the 2,6DNT regulated gene network, the genes CDKN1A, PAK1, ITGAV, and NFE2L2 (NRF2) were highly connected (Figure 9C) suggesting these genes are important in 2,6DNT effects on liver. Interestingly, the highly connected genes in the TNT regulated gene network (Figure 9F) were largely involved in immune response. These genes consisted of CXCL10, CCL13, TNFSF10, NOS3 and CCND1 and may play a major role in TNT adverse effects on the liver.

In the 2ADNT affected gene network, CDKN1A and ABCC3 were greatly connected (Figure 9D), indicating possible roles in 2ADNT affected potential liver function. Genes including HMOX1, PRKCz, PKC, Raf, and RAF1 were highly connected in the gene network responsive to 4ADNT (Figure 9E). These genes could play more important roles in potential liver effects regulated by 4ADNT.

Despite the different topologies and gene compositions of the networks, a number of common pathways that were modulated by all compounds and were also present in all sub networks. These included NRF2-mediated oxidative stress response, aryl hydrocarbon receptor signaling, xenobiotic metabolism signaling, LPS/IL-1 mediated inhibition of RXR function, and xenobiotic metabolism signaling pathways.

### Verification of microarray responses using real time QRT-PCR

To verify the credibility of microarray data, we first selected 96 genes that are responsible for oxidative stress (Table S12) to perform real time quantitative reverse transcriptase PCR (QRT-PCR). Real time qPCR studies were conducted using vehicle control samples and 199 mg/kg 2,4DNT samples at either 24 or 48 h time points. The same RNA was used for QRT-PCR as was used for microarray assessment. We found a surprising consistency between the two approaches. The significantly regulated gene numbers by 2,4DNT were exactly the same for both QRT-PCR and microarray, 32 for 24 h and 34 for 48 h (File S1). Moreover, both methods were identical for the positive and negative directions of all the genes between both time points (File S1). A regression coefficient of 0.93 between the two approaches for both time points was achieved (File S1).

Previous studies by Wintz et al.[2] found that 24DNT impacted oxygen transport and lipid metabolism in livers of the fish fathead minnow (Pimephales promelas) via interaction with hemoglobin and peroxisome proliferator activated receptor alpha (PPARα) pathways. We examined whether similar impacts are observed in rats exposed to nitrotoluenes. To further verify gene expression profiles, genes peroxisome proliferator activated receptor alpha (PPARα), transferrin (TF), apolipoprotein B (APOB), and alpha Hemoglobin (aHG) were chosen to carry out QRT-PCR across all samples including different compounds, doses, and time points (File S1). Correlations of 0.71 and 0.85 were observed between the results of QRT-PCR and microarrays (File S1). In fathead minnows, the gene TF was down-regulated in livers of fathead minnows exposed to 24DNT. Here, expression of TF was also attenuated by 2,4DNT at 24 h, in addition to 2,6DNT at 24 and 48 h with clear dose responses for both QRT-PCR and microarray with R^2^ = 0.79 (File S1). The gene PPARα has been shown to be repressed by 2,4DNT in liver of exposed fathead minnow. PPARα was also down-regulated by 2,4DNT at 24 h in rat with a strong dose response. PPARα was consistently suppressed by 2,6DNT at 24 h and 48 h but up-regulated by 2ADNT and TNT at 48 h. Microarray and QRT-PCR data had an R^2^ = 0.84. Other genes such as APOB were reported to be down-regulated in fathead minnows exposed to 2,4DNT while alpha Hemoglobin (aHG) was up-regulated. Hepatic nuclear factor 4, alpha (HNF4A) was not affected by 2,4DNT in fathead minnows. HNF4A had no significant change with exposure to the five chemicals (not shown).We saw similar impacts on these 3 genes by 2,4DNT in rat livers by both QRT-PCR and microarray (File S1). While APOB was up regulated by all chemicals, aHG was up regulated by 24DNT but it was primarily down-regulated by other compounds (File S1). Microarray and QRT-PCR data for APOB and aHG had R^2^ values of 0.75 and 0.71 respectively (File S1).

### QRT-PCR analysis of genes found in networks and affected pathways

QRT-PCR analysis of select genes present in gene networks and affected pathways were performed including GSTM4, NRF2, ALDH1A1, and fatty acid binding protein 2 (FABP2) had R^2^ values of 0.85, 0.83, 0.73, and 0.72, respectively, when compared to microarray data across all samples (File S1). Differential effects were seen on NRF2 expression with up-regulation at 24 h with 2,4DNT and 2,6DNT while it was down-regulated at 24 h with 2ADNT, 4ADNT and TNT. At 48 h, NRF2 expression was up-regulated with 2,4DNT, 2ADNT, 4ADNT and TNT while no significant effect was seen with 2,6DNT exposure. GSTM4 expression was consistently up regulated at 24 h by all five chemicals. Differential effects due to nitrotoluene exposure were observed on expression of ALDH1A1. 2,4DNT up-regulated ALDH1A1 at both 24 and 48 h. 2,6DNT down-regulated ALDH1A1 at 24 h while producing little consistent effect at 48 h. 2ADNT up-regulated expression at 24 h and down-regulated expression at 48 h. 4ADNT up-regulated expression at 24 and 48 h. TNT had a slightly inconsistent effect but generally up-regulated ALDH1A1 expression at both 24 h and 48 h. Fatty acid binding protein 2 (FABP2) was up-regulated by all chemicals except 2,6DNT which only up-regulated FABP2 at a single dose and time.

### Nitrotoluene exposure adversely impacts lipid metabolism

**Figure 10:**
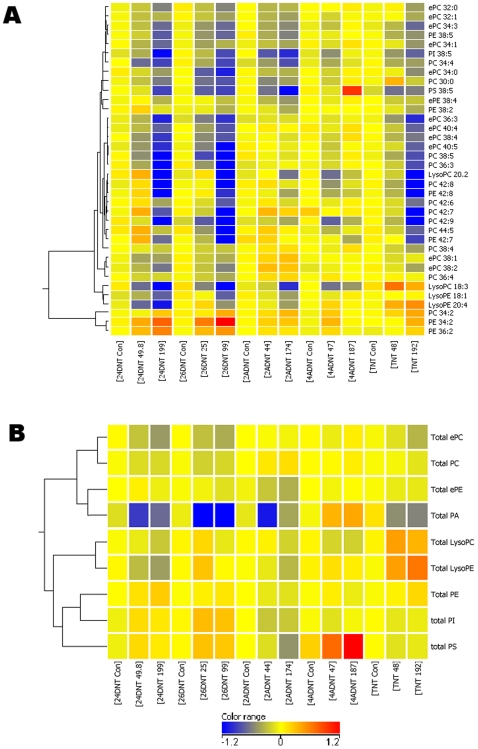
Hierarchical clustering of commonly significantly regulated lipid species in response to 2,4DNT, 2,6DNT, 2ADNT, 4ADNT and TNT. Rats were treated with 2,4DNT, 2,6DNT 2ADNT, 4ADNT or TNT with one of 3 doses including controls for each compound for 24 h. Rats were sacrificed and liver tissues were used for lipid extraction and lipid profile measurement as described in Materials and Methods. Actually the same samples were also used for the microarray studies. 36 individual lipid species (A) and 9 total lipid classes (B) that were significantly commonly regulated by at least 3 of the compounds. Significantly regulated individual lipid species or lipid classes were determined using One-Way ANOVA with a multiple adjust p value less than 0.05 across various doses of a compound.

The enrichment in GO biological process related to lipid metabolism and pathways including fatty acid metabolism and NRF2-mediated oxidative stress response in addition to data from other species suggests that nitrotoluenes exposure would significantly impact lipid biosynthesis in liver. To validate this we analyzed lipid metabolites in livers of the exposed rats. Two doses were selected at 24 h to measure lipids levels, the second lowest and the highest, and vehicle control of the 5 compounds. A total of 340 lipid species (Table S13) belonging to 12 lipid classes were measured using a mass spectrometry approach [19], [20]. One-Way ANOVA with a multiple adjustment (p value < = 0.05) was applied to identify differentiated lipid species for each compound. 2,4DNT, 2,6DNT and TNT had stronger impact on the regulation of lipid profiles than 2ADNT and 4ADNT. Overall, more lipid species were down-regulated by the compounds. Thirty-six lipid species were commonly affected by at least 3 of the 5 compounds (Figure 10A). Of them, 21 lipid species were commonly down-regulated in response to 2,4DNT, 2,6DNT and TNT: Nine PC with one ether-linked (alkyl or alkenyl) chain (ePC), 8 phosphatidylcholine (PC), and 4 phosphatidylethanolamine (PE). Phosphatidylinositol (PI 38∶5; phospholipid species are indicated by total acyl carbons∶total acyl carbob-carbon double bonds) and lysoPC20∶2 were down-regulated by all the compounds. Only 3 lipid species, PC 34∶2, PE 34∶2 and PE 36∶2 were up-regulated by all the compounds. The lipid species lysoPC 18∶3 was decreased by 2,4DNT, 2,6DNT, 2ADNT and 4ADNT but increased by TNT. PS 38∶5 was down-regulated by 2,4DNT, 2,6DNT, 2ADNT, and TNT but up-regulated by 4ADNT. Interestingly, no matter whether a lipid species was up or down-regulated, a strong dose effect was observed (Figure 10A). We also compared total lipid species differences in each lipid class. There were more total lipids that were down-regulated by 2,4DNT, 2,6DNT, 2ADNT and TNT except 4ADNT (Figure 10B). Total ePC, ePE were commonly down-regulated by all the compounds. There was no single lipid class that was commonly up-regulated by all 5 compounds. The amount of total PA was reduced in response to 2,4DNT, 2,6DNT, 2ADNT and TNT but was elevated in response to 4ADNT. Total PS was up in response to 2,4DNT, 2,6DNT and 4ADNT but down to 2ADNT and TNT. Lipidomics results of an overall down-regulated lipid profile are well correlated with the gene expression profiles where lipid metabolism was predicted to be inhibited.

## Discussion

In the present study, we have used a systems biology approach in order to understand effects of the 5 similarly structured nitrotoluenes on signaling pathways via liver gene alteration and to gain insight into the mechanism of toxicity of this class of compounds. An integrative approach was employed in our study which included clinical toxicology, pathology, transcriptomics, lipidomics, gene function classification, pathway analysis and gene network modeling. Overall, we found that the expression results correlated well with toxic and pathological results. For instance, we have found more genes that were significantly changed in response to 2,4DNT and 2,6DNT than TNT, 4ADNT, and 2ADNT. Interestingly, we found that the number of regulated genes was greater at 24 h than 48 h by all compounds except TNT. Animals might take more time to metabolize TNT to an unidentified reactive metabolite, which could be a reason why TNT impacts more genes at 48 h than 24 h. The difference in the amount of differentially expressed genes, distinctive gene expression patterns, specific regulated gene lists, pathways and unique gene networks, suggest a specific molecular mechanism for each compound. Although we observed clinical and histo-pathological differences caused by the compounds, we could still see some common biological processes that were affected by two or more compounds, which involve DNA damage response and cell death signaling, detoxification response, de-regulation of lipid metabolism and impaired immune response.

### DNA damage response and cell death signaling

Chemicals, irradiation, or other environmental factors can induce DNA damage response which results in cell death via cell cycle arrest [21]. Several earlier studies reported that 2,4DNT and 2,6DNT could cause cell death and genotoxic effects such as DNA damage [22]. TNT and its metabolites have also been determined to induce DNA fragmentation and damage [23]. 2,4DNT was more potent than TNT in inducing DNA single strand breaks in hepatocytes from livers of rats treated orally [24]. TNT and 2,4DNT could also possibly alter DNA sequence [25].

Our study has revealed that DNA metabolic process and DNA damage response are major biological processes affected by these compounds, which seem to be more significant with 2,4DNT, 2,6DNT and 4ADNT than TNT and 2ADNT. One possible reason for the damage caused by these compounds is the ability to impair DNA synthesis and replication through DNA adduction as was discovered in hepatocytes exposed to 2,4DNT and 2,6DNT [26]. Another potential reason is through endogenous oxidative processes which are known to induce DNA damage [27]. Oxidative process is covered in detail in the next section. The DNA damage activates ataxia telangiectasia mutated (ATM) kinase and other kinases, leading to the activation of the tumor suppressor p53 [28]. P53 is a transcription factor that has long been recognized to play a key role in many biological processes such as cell cycle, DNA repair, cell death and apoptosis [29], [30].

Cell death signaling was also found to be induced by these compounds. For instance, caspase 3, known to induce cell shrinkage and DNA fragmentation that leads to cell death, was up-regulated by the compounds [31].

### Oxidative stress and detoxification response

One important pathway that could play a role in oxidative stress and detoxification response is the NRF2-mediated oxidative stress response pathway, which was commonly influenced by all the 5 compounds. NRF2 is a transcription factor which binds to the antioxidant response element in the promoter of NRF2 regulated genes [32]. Under normal conditions NRF2 is bound to Keap1 in the cytoplasm [33]. A number of factors such as free radicals reverse the sulfhydryl-dependent association between NRF2 and KEAP1 and release NRF2, allowing it to enter into the nucleus.

The NRF2-mediated oxidative stress response has been revealed to reduce toxicity and carcinogenesis during exposure to electrophile or other environmental toxicants or inflammation in mammalian models [34]. Recently, we found that NRF2 was also induced in the liver of Northern bobwhite (*Colinus virginianus*) treated with 2,6DNT [35] suggesting NRF2 mediated pathway is functionally conserved across different species for the protection from 2,6DNT.

Several NRF2 downstream phase I and II metabolizing enzymes including GSTs (GSTM3, GSTM4, etc.), NQO1, Aflatoxin B 1-aldehyde reductases (AKR7A3), epoxide hydrolase 1, microsomal (xenobiotic) (EPHX1) and UGT1A6 were significantly induced by most of the compounds and may prevent cell damage due to oxidative stress.

Another key pathway contributing to the detoxification process is aryl hydrocarbon receptor (AHR) Signaling. AHR ligands bind to the AHR complex inducing the dissociation of interacting proteins, leading to the release of AHR which then complexes with the aryl hydrocarbon receptor nuclear translocator (ARNT) and facilitates transfer of the complex to the nucleus and subsequent regulation of gene expression leading to biochemical and toxic responses [36]. AHR signaling has been also reported to play a role in detoxification [37]. AHR regulates expression of phase I metabolizing enzymes in the aldehyde dehydrogenae (ALDH) families. Two key members in this family, ALDH1A1 and ALDH1L1, were significantly up-regulated by nearly all the compounds. ALDH family genes have been well characterized as protectors ROS caused oxidative damage. They metabolize reactive products of toxic lipid peroxidation (LPO), which include 4-hydroxy-2-nonenal and malondialdehyde [38]. This could explain why ALDH could contribute to the detoxification of the toxicity induced by the compounds. ALDHs are also well-known nitroreductases.

The expression of many CYPs downstream of AHR signaling is significantly regulated by these compounds, which include CYP1A2, CYP3A5, CYP3A43, CYP4A11, CYP7A1, CYP51A1, CYP2C7, CYP2C44, CYP2C70, CYP2D6, CYP2D12, CYP2D26, CYP4F8, and CYP2J9 etc. An induction of CYPs via AHR signaling is to enhance clearance of these compounds. However, if a compound is present in excess it will form an electrophilic nitrenium cation that will adduct nucleophiles of DNA, e.g., guanine, and proteins, e.g., hemoglobin as studied by Sabbioni et al.,[39].

Since these commonly regulated pathways such as NRF2-mediated oxidative stress response, aryl hydrocarbon receptor signaling and metabolism of xenobiotics by cytochrome P450 pathways are part of xenobiotic metabolism signaling, xenobiotic metabolism signaling may play a pivotal role in the protection against the toxicity from the compounds. A variety of phase I and phase II metabolizing enzymes belonging to several gene families including GSTs, CYPs, ALDHs, UGTs, and aflatoxin B1 aldehyde reductase (AFARs) seem to participate in all the xenobiotic pathways.

In addition to NRF2, AHR, and P450 mediated pathways, the constitutive androstane receptor (CAR) and the pregnane x receptor (PXR) mediated pathways are part of xenobiotic pathway as well [40]. ATP-binding cassette, sub-family C (CFTR/MRP), member 3 (ABCC3), is a transporter protein and downstream target for both CAR and PXR signaling, and its expression was commonly elevated by the compounds and likely serves as a role in the defense against the toxicity caused by the compounds [41].

GO analyses revealed that differentially expressed gene lists were highly enriched in the biological process “protein folding” by all the compounds. HSPs are well known stress response proteins involved in multiple functions such as protein folding and unfolding, cell survival and cell growth [42], [43]. The expression of HSPs, was significantly enhanced by all or some of the compounds. HSPs also participate in xenobiotic metabolism signaling pathway. Therefore, their induction by the compounds should also contribute to the detoxification process.

### Nitrotoluene effects on lipid metabolism

GO enrichment analyses suggested that “lipid metabolic process” was a process significantly repressed by almost all the compounds. Pathway analysis also found that two pathways with major roles in lipid metabolism were affected: LPS/IL-1 mediated inhibition of RXR function and fatty acid metabolism. Chemical analysis of livers from these compounds exposed rats for 24 h confirmed genomics evidence that lipids were adversely affected including individual lipid species and total lipid classes. Down-regulation of lipid metabolism associated genes leads to the reduction of lipid metabolite products and possible energy production.

An important part of LPS/IL-1 mediated inhibition of RXR function is PPAR/RXR signaling. Fatty acid oxidation in liver and other tissues is regulated via the activation of nuclear hormone receptors, including PPARs and FXR [44], [45]. These nuclear hormone receptors interact with RXR to activate gene transcription. Recent studies have revealed that the expression of RXR, PPARα, are decreased in liver, kidney, and heart after the treatment of lipopolysaccharide (LPS) [46]–[48]. Both qPCR and microarray results (File S1) show that PPARα was repressed by 2,4DNT and 2,6DNT, which was observed in fish [2].

A target of PPAR/RXR signaling, Acyl-CoA synthetase ACSL5, was strongly down-regulated by 2,4DNT and 2,6DNT. Other members Acyl-CoA synthetases such as ACSL1, ACSL2, ACSL3 and ACSL4 were also affected by 2,4DNT and to some extent 2,6 DNT. Acyl-CoA synthetases catalyze long chain fatty acids to acyl-CoAs. Acyl-CoAs have a variety of metabolic fates in the cell and can be employed to acylate proteins or be metabolized through catabolic pathways such as β-oxidation or anabolic pathways such as *de novo* synthesis and reacylation of triacylglycerol (TAG), phospholipids, and cholesterol esters [49]. Acyl-CoAs have also been reported to be involved in cell survival and lipid uptake, therefore inhibition of Acyl-CoAs could affect cell survival and normal lipid uptake [49].

In contrast to Acyl-CoAs, fatty acid binding protein 2 (FABP2), another downstream target of PPAR/RXR signaling [50], [51] was induced by almost all the compounds. Fatty acid binding proteins participate in the uptake, intracellular metabolism and/or transport of long-chain fatty acids. They may also be responsible in the modulation of cell growth and proliferation. Under normal situations, fatty acid binding proteins bind to endogenous fatty acids in the cytosol in mammals [52] enabling regulation of fatty acid metabolism and maintenance of free fatty acids at less than toxic concentrations [53]. Some recent studies have shown that certain chemicals such as perfluorinated compounds (PFCs) can dissociate the binding of FABPs to fatty acids by displacing them from the protein [52] making them free to facilitate the transcription of FABP mRNA [54]. FABP2 was up-regulated by nitrotoluenes possibly due effects similar to those found for PFCs. In contrast, FABP2 is down-regulated by 2,4DNT in fish [2], indicating a species specific mechanism for 2,4DNT impacted lipid metabolism.

LIPC, FASN, and APOB are primarily downstream genes of FXR/RXR signaling, which is also a part of LPS/IL-1mediated inhibition of RXR function pathway. These genes were more heavily repressed by 2,4DNT and 2,6DNT. Lipase, hepatic (LIPC) is mainly involved in lipoprotein transport and metabolism of HDL while FASN plays a major role in fatty acid synthesis. The down-regulation of APOB supported by both qPCR and microarray is in agreement with the observation in fish exposed to 2,4DNT. The reduction of the expression of these genes could impair normal lipid metabolism. Another recent study by our laboratory has shown that FASN and APOB were also down-regulated in the liver of Northern bobwhite (*Colinus virginianus*) treated with 2,6DNT indicating universal biomarkers for impaired lipid metabolism could be conserved across different species for the compounds [35].

### Immune response

Immune response is the most significant functional term for those down-regulated genes by 4ADNT and TNT. The major histocompatibility (MHC) class I E (HLA-E) is a key member of MHC class I. Its expression was repressed by almost all the compounds. HLA-E is a ligand for receptors of both the adaptive and innate immune systems. The binding of self-peptides complexed to HLA-E by the CD94-NKG2A receptor of natural killer (NK) cells is a critical checkpoint for immune surveillance by NK cells. Additionally, HLA-E is recognized by the T-cell receptor of alpha/beta CD8 T cells as well and contributes to the adaptive immune response to invading pathogens or other xenobiotics[55]. The reduction of HLA-E expression by the compounds indicates they could disrupt the normal immune protection system and induce abnormal phenotypes such as inflammation or other diseases.

Chemokine signaling is a major immune response that was affected by the compounds. It seems different compounds may target different chemokine signaling pathways. Chemokine (C-X-C motif) ligand 12 (CXCL12 or SDF-1) was strongly down-regulated by 2,4 DNT and 2,6DNT. It is a ligand for the G-coupled receptor protein Chemokine (C-X-C motif) receptor CXCR4. Activation of CXCR4 by CXCL12 is involved in many biological functions such as cell migration, growth and survival. The activation can also trigger leukemia cell movement to the marrow microenvironment, where CXCL12 pushes leukemia cells in close contact with marrow stromal cells that lead to growth and drug resistance signals. The inhibition of the expression of CXCL12 could make the cells more susceptible to the nitrotoluenes. It seems there are many chemokine ligands that were repressed by TNT, which include CXCL13, CXCL9, CXCL10, etc. Interestingly CXCL9 and CXCL10 share the same G-protein coupled receptor CXCR3. Therefore, these chemokines possess the capability of maintaining the normal immune function and the protection against infectious agents and other external toxicants [56], [57]. CXCR3 was significantly down-regulated, particularly by TNT but whether CXCR3 is a direct target of TNT needs to be further examined.

### Correlation of gene expression and lipid profiles with adverse effects

Our toxicity studies have shown that overall dinitrotoluene compounds, 2,4DNT, 2,6DNT and TNT had stronger toxic effects than amino-dinitrotoluene compounds 2ADNT and 4ADNT, among which it seems that 2,4DNT and 2,6DNT are more toxic than TNT. For example, we only observed lethality of rats with 2,4DNT and 2,6DNT treatments. Another unique effect of 2,4DNT, 2,6DNT and TNT exposure was hepatic sinusoidal congestion. We found that erythrocytosis was associated only with dinitrotoluenes 2,4DNT and 2,6DNT. These results were reflected by the overall number of genes whose expression was changed. 2,4DNT, 2,6DNT had more gene expression changed than TNT, 2ADNT and 4ADNT. Our gene expression analysis also showed that 2,4DNT and 2,6DNT share more similar gene expression pattern than the other compounds.

At 48 h, TNT affected more changed gene numbers than 2ADNT and 4ADNT. 2ADNT had the least change in gene number, which may be linked to its weak effects on body and normalized liver weight and no change in RBC parameters or blood granulocytes.

We found that body weight gain was decreased by all the compounds. One probable reason for this observation was the reduction of food consumption due to nitrotoluene exposure. A reduction in food consumption may also play a role in the observed down-regulation of energy metabolism. Our gene expression and lipid profiles clearly show that energetic related lipid metabolism is decreased, which supports this statement.

Liver weight increase, as seen in rats exposed to TNT and 4ADNT at 48 h, is indicative of enzyme induction including cytochrome P450s and GSTs. Many of these enzymes were induced at both 24 h and 48 h by the nitrotoluenes. Lipid metabolism especially fatty acid metabolism, [58] and PPAR signaling are associated with liver weight increase [59]. The gene expression did show that fatty acid metabolism and PPAR signaling were affected by the compounds. Mounting evidence has shown that ROS can cause liver weight increase [59], [60], and our gene expression results indeed implicate ROS could be produced by these compounds.

The most remarkable effect we observed from the hematology assessment was erythrocytosis induced by the dinitrotoluenes. Even though we did not find anemic effects induced by 2,4DNT as have been reported in other studies, we did see decreased hemoglobin and hematocrit in 4ADNT exposed animals after 48 h. One possible reason why anemia was not observed in our studies is that we conducted an acute exposure design; the other studies where anemia was seen are all repeat dose studies.

In our study, expression of several genes related to erythrocytosis and oxygen transport such as hemoglobins and transferrin were affected by the dinitrotoluenes. Numerous blood parameters were unchanged or decreased in contrast to the hematological parameters associated with erythrocytosis. This suggests that the latter did not result from hemoconcentration as might occur if dehydration occurred.

By purposely looking at erythropoietin signaling genes such as erythropoietin and its receptor, we found that their expression was also altered. Hemoglobin adduct formation due to nitrotoluene exposure has been reported and may adversely affect oxygen transport [39]. Hematotoxicity could be the direct toxicity of these compounds followed by sinusoidal congestion and hepatoxicity such as, necrosis, inflammation, toxicities to other organs and even lethality because the delivery of oxygen to tissues is impaired. Although extensive hepatotoxicity was not generally observed in our short experimental design, some evidence such as the sinusoidal congestion in histopathology after 2,6DNT, 2,4DNT and TNT treatments and liver toxicity related gene expression could predict more serious hepatoxicity with longer and continued exposure. That hepatotoxicity might ensue as a time-dependent secondary effect of hematotoxicity is supported by the observation [61] that mechanism of action of dinitrotoluenes on cytotoxicity of isolated rat hepatocytes in culture differs from any of the pathways implicated by gene expression changes in our whole animal exposures.

Hypoxia signaling plays an important role in erythrocytosis [62]. Interestingly, we found that many genes in this pathway were up-regulated by 2,4DNT, 2,6DNT and TNT. For instance, casein kinase 1, delta(CSNK1D), HSP90AA1, AP-1, MDM2, ubiquitin conjugating enzyme E2D2 (UBE2E2), UBE2M, UBE2N and P53 were induced by 2,4DNT (File S1). The presence of hypoxia could also induce ROS production, inflammation, oxidative stress and inflammation.

### Integrative model for the common mechanisms

**Figure 11:**
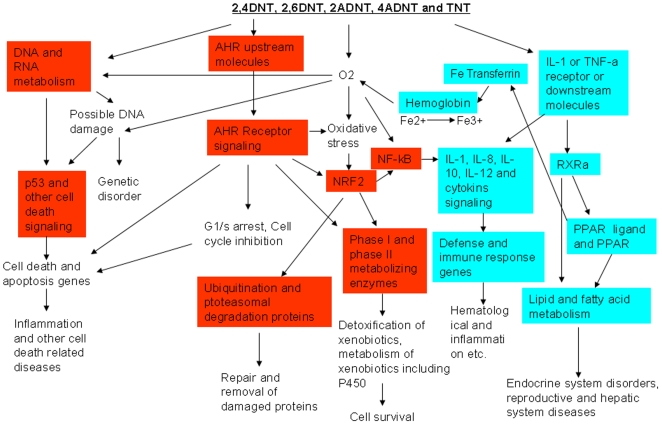
An integrated network model to explain the molecular mechanisms of hepatoxicity induced by the compounds. Environmental chemicals, gene functional terms, pathway cross talks, gene networks, and physiological endpoints were integrated to form a network at the system level to account for the molecular mechanisms of hepatoxicity mediated by these compounds. Red and green highlighted functional terms, genes, and pathways were generally up-regulated and down-regulated respectively by these compounds.

An integrative analysis of physiological endpoints, gene ontology, pathways, and gene networks across different aspects we were able to determine a possible mechanism of liver toxicity due to exposure of these nitrotoluenes (Figure 11). DNA damage response is a major functional term under DNA metabolic process and is possibly due to direct damage by the chemicals or reactive oxygen species (ROS), which are produced as side products of phase I metabolizing enzymes such as CYPs. These enzymes are mainly under the functional term protein process, and are involved in the xenobiotic metabolism signaling pathway. The protein PPARα, controls expression of lipid metabolism genes (apolipoproteins and fatty acid metabolism genes) as well as transferrin (TF) gene. Similar to the observation in fish treated with 2,4 DNT, the expression of TF was primarily decreased by these compounds (File S1). Hemoglobin expression in liver tissue was weakly increased by 2,4DNT, and interestingly it was reduced by 2,6DNT at 24 h and elevated at 48 h, and was largely reduced by 2ADNT, 4ADNT and TNT. Transferrin carries iron, which is a critical cofactor of hemoglobin. Oxygen is a substrate of hemoglobin. 2,4DNT can influence oxygen transport by oxidizing hemoglobin ferrous iron to its ferric state [2]. Hence, oxygen transport gene deregulation can induce hematological disorder including erythrocytosis.

DNA response activates P53 and other cell death signaling which ultimately leads to inflammation and other cell death related diseases. AHR signaling also affects cell death by regulating P53 signaling and controlling cell cycle gene expression. Two pathways, AHR and NRF2, belonging to xenobiotic pathway are primarily responsible for protecting against the toxicity induced by the compounds. Activation of downstream phase I and phase II enzymes in these two pathways contribute to detoxification of xenobiotics leading to cell survival. Another critical commonly regulated pathway is LPS/IL-1 mediated inhibition of RXR function pathway. PPARs such as PPARa and PPARg are involved in this pathway and regulate downstream molecules contributing to lipid metabolism which is an important significant common function term down-regulated by all the compounds, as well as fatty acid metabolism that is a commonly regulated pathway. The reduction of lipid metabolism by the compounds could result in endocrine system disorders, reproductive and hepatic system diseases. LPS/IL-1 mediated pathway is certainly connected with interleukins and cytokines mediated signaling which play a role in defense and immune response, which was a common functional term more heavily down-regulated by TNT and 4ADNT. Inhibition of immune function by the compounds could lead to inflammation related diseases. The production of ROS can also activate NF-kB that connects to ILs and cytokine signaling. The P53 pathway could also connect with hypoxia signaling and play a pivotal role in erythrocytosis (File S1). We found that dinitrotoluene compounds, 2,4DNT, 2,6DNT and TNT had stronger toxic effects than amino- dinitrotoluene compounds 2ADNT and 4ADNT. There are some specific pathways such as nucleotide excision repair pathway that were only significantly regulated by 2,4DNT and 2,6DNT which could explain why these two chemicals induce heavier adverse effects. Immune response related antigen presentation pathway was only evidently affected by TNT, and there are also more chemokine ligands that were repressed by TNT. From this, it appears that the immune system is more of a target for TNT than the other 4 nitrotoluenes.

We also found that liver toxicity could be a secondary effect of primary hematological toxicities caused by these compounds. We found hypoxia signaling could be an important pathway affected by the compounds.

### Conclusions

The present study is the first *in vivo* toxicogenomics study to investigate the gene transcriptional responses of these munitions compounds in mammals. We found that 2,4DNT, 2,6DNT and TNT had stronger toxic effects than 2ADNT and 4ADNT. Xenobiotic metabolic process was commonly activated by the compounds and lipid metabolism and immune response were commonly repressed by the compounds. Several pathways such as NRF2-mediated oxidative stress response, aryl hydrocarbon receptor signaling, LPS/IL-1 mediated inhibition of RXR function, xenobiotic metabolism signaling and metabolism of xenobiotics by cytochrome P450 were commonly regulated by the chemicals.

Our results indicate that an integrative systems biology approach along with physiological findings is an efficient approach to gain insight into both individual and common mechanisms of action of the nitrotoluenes in a mammalian system. Concepts presented in this study can be utilized by others in their research to understand how a toxicant produces its toxicity.

## Materials and Methods

### Chemicals

2ADNT (99.9%) and 4ADNT (99.9%) were obtained from SRI International (Menlo Park, CA). TNT (99.9%) was obtained from Eastman Chemical Company (Kingsport, TN). 2,4DNT (97%) and 2,6DNT (98%) were obtained from Sigma-Aldrich (St. Louis, MO).

### Animals and Treatment

Female Sprague-Dawley rats (175–225 grams) were from the in-house breeding colony (College of Pharmacy, University of Louisiana at Monroe [ULM] and treated in accordance with the *Guide for Use and Care of Animals* [63]. Breeders were from Harlan-Sprague Dawley in Madison, WI. Housing consisted of a 12 h light/dark cycle with *ad libitum* access to tap water and rodent chow (Harlan/Teklad 7012, Madison, WI). Rats were housed individually in polycarbonate cages on hardwood bedding (Sani-chips, Harlan/Tekland, Madison, WI) one week prior to treatment. Food was withdrawn the night before treatments, which were administered by gavage between 8 and 10 AM. Study protocols were preapproved by the Institutional Animal Care and Use Committee of the University of Louisiana at Monroe (Animal Welfare Assurance Number A3641-01).

Groups of rats were weighed and randomly assigned to treatment. Treatments were vehicle (5% v/v DMSO in corn oil), TNT (4.8, 48, 96, 192, and 384 mg/kg), 4ADNT (4.7, 47, 94, 187, and 374 mg/kg), 2ADNT (4.4, 44, 87, 174, 348 mg/kg), 2,4DNT (5, 50, 99, 198 and 398 mg/kg), and 2,6DNT (5.0, 25, 50, 99, 199, 398 mg/kg). Rats were observed continuously for the first hour after dosing, hourly for 8 h and daily thereafter. Moribund rats were euthanized with CO_2_. All animals died when dosed with the 2,6DNT high dose (398 mg/kg). At 24 or 48 hours after treatment, survivors were anesthetized with CO_2_ and the sternum was bisected to expose the heart. Blood was collected by cardiac puncture of the left ventricle into EDTA-containing Vacutainer tubes (Becton, Dickinson and Co., Franklin Lakes, NJ) for hematological assessment. Serum was derived from blood drained from the heart after nicking the ventricles. Livers were excised and weighed. A 2 mm slice from the medial lobe was fixed overnight in neutral buffered formalin, then 70% ethanol. A portion of the liver was removed and placed in RNA Later (Ambion) following manufacturer's instruction and later used for genomic analyses. Remaining liver was flash frozen in liquid N_2_ and stored at −70°C for lipidomic and analytical chemistry analyses.

### Analytical Chemistry

Upon sacrifice, a portion of liver was flash frozen with liquid nitrogen then transferred to −80°C. Samples were thawed and weighed in bead beater tubes (MP Bio, Solon, OH). Next, 500 µL of high performance liquid chromatography (HPLC)–grade acetonitrile (>99.93%) (Sigma-Aldrich, St. Louis, MO, USA) was added to each bead beater tube before being homogenized using a FastPrep®-24 Instrument (MP Bio, Solon, OH, USA). After lysing, samples were centrifuged at 16,500 g in a microcentrifuge to pellet debris. Each supernatant was then diluted at a ratio of 1∶1 with organic free reagent water. Each diluted extract was then added into a 200 µL glass insert placed in a 1.5 mL amber vial (Agilent Technologies, Santa Clara, CA, USA). Samples were analyzed by HPLC using methods modified from Johnson et al. [64]. Any concentration <0.200 mg/kg was considered an estimated value because it was below the lowest calibration standard. Nitrotoluene standards were analyzed before during and after sample analysis with 90–110% recovery to ensure accuracy.

### Toxicological Assessments

Histopathological assessment of livers were performed on sections (5 µm) cut from tissues embedded in paraffin and stained with hematoxylin and eosin (H&E) using routine procedures [65]. Hematological endpoints were determined by a clinical laboratory at a local hospital with a CELL-DYN Sapphire System (Abbott Laboratories, Abbott Park, IL) [66]. Neutrophils, eosinophils and basophils were summed and reported as granulocytes. Reticulocytes in peripheral blood were vitally stained with new methylene blue according to manufacturer's instructions (Procedure No. R 4132, Sigma-Aldrich, St. Louis, MO). Wedge preparations of stained blood were prepared and slides coded. Reticulocytes were determined as percentage of 2000 red blood cells counted per animal.

Serum was analyzed for a panel of metabolic parameters by the hospital clinical laboratory with an Abbott Architect Ci 8200. Absolute values for albumin of vehicle-treated rats were low relative to published normal values [67], which were most likely due to use of the bromocresol purple binding method with the instrument calibrated for human albumin. Non-human albumin has been shown to be less reactive with this dye [68]. Values for the serum transaminases were approximately twice published normal values probably due to interference with the colorimetric assay since serum samples collected from the nicked heart exhibited some hemolysis as evidenced by a pink color. Hemolysis was not of such an extent however to affect MCHC. Contamination with enzyme from nicked tissue may also have contributed to these values. However, these were not expected to affect detection of treatment related differences because all trials included concurrent vehicle-treated control groups.

### Statistical Analyses

Clinical endpoints were analyzed for homogeneity of variance with Levene's test and no heteroscedasticity was observed. Data were thus analyzed with ANOVA; post-hoc comparisons of treatment means against concurrent vehicle control were done with Dunnett's test. A square root transformation with 3/8 continuity factor was performed on reticulocyte percent data before analysis. Treatment mean differences from control with p<0.05 were considered significant.

### Microarray experimental design

Changes in gene expression were tested using Agilent commercial whole rat genome microarrays (4×44K). Experiments were designed for both purposes of temporal and dose response analysis. For temporal analysis, only two time points for each compound were used, 24 h and 48 h. For dose response analysis, four doses plus a vehicle control were employed for each compound at each time point. A separate control was used for different compounds even at the same time point. The dose selection was based on the LD 50 data for each compound. Four biological replicates of this design were conducted, each using different animals, and four biological replicates were applied, which resulted in a total of 200 microarrays.

### Total RNA extraction

Total RNA was extracted from about 30mg of liver tissue. Tissues were homogenized in the lysis buffer with FAST Prep-24 from MP before using RNeasy kits (Qiagen). Total RNA concentrations were measured using NanoDrop® ND-1000 Spectrophotometer (NanoDrop technologies, Wilmington, DE, USA). The integrity and quality of total RNA was checked on an Agilent 2100 Bioanalyzer (Palo Alto, CA). The gel-like images generated by the Bioanalyzer show that total RNAs have two bands, represent 18S and 26S RNA of mammalian RNA. Nuclease-free water (Ambion) was used to elute total RNA.

### Microarray hybridization

Rat whole genome oligo arrays in the format of 4X44K were purchased from Agilent Technologies. Sample cRNA synthesis, labeling, hybridization and microarray processing were performed according to manufacturer's protocol “One-Color Microarray-Based Gene Expression Analysis” (version 1.0). The labeling reactions were performed using the Agilent Low RNA Input Linear Amplification Kit in the presence of cyanine 3-CTP. The labeled cRNA from each labeling reaction was hybridized to individual arrays at 65°C for 17 h using Agilent's Gene Expression Hybridization Kit. After washing, the arrays were scanned using a GenePix 4200AL scanner (Molecular Device Inc.). The Feature extraction software (V. 9.5.1) from Agilent was used to automatically find and place microarray grids, reject outlier pixels, accurately determine feature intensities and ratios, flag outlier pixels, and calculate statistical confidences.

### Microarray data analysis

Microarray data analyses were processed with GeneSpring version 7.0 and 10.0. The sample quality control was based on the Pearson correlation of a sample with other samples in the whole experiment. If the average Pearson correlation with other samples was less than 80%, the sample was excluded for further analysis. If the scanned intensity was less than 5.0 for a probe, it was transformed to 5. Only one liver sample, TNT at 4.8 mg/ml for 24 h, did not pass the criteria and was excluded. A perchip (within) array normalization was performed using 50 percentile values of all the probe values in the array. Per gene (between) array normalization was also applied using the median value of a gene across all samples in the experiment. Probe features were first filtered using flags. A “present” or “absent” flag was defined using the Agilent *Feature Extraction 9.5.1* software. Only a probe that had present flags in at least 50% samples of all the arrays was kept for further analyses. Data were subsequently log (base 2) transformed for statistical analyses. Microarray data has been deposited in the GEO database (GSE19628).

To extract only significantly regulated genes, a One-Way ANOVA was performed across 4 doses and a matched control sample for one compound at a time at either 24 h or 48 h. Benjamini and Hochberg False Discovery (BHF) Rate with a FDR [69] was applied and only the transcripts with FDR-adjusted P value < = 0.05 were considered significant differences. Meanwhile, a Tukey post-hoc test was conducted to compare the 5 sample groups including comparing the control group with any one of treatment group at a certain dose within a One-Way ANOVA test.

To compare the overall gene expression profiles induced by the 5 compounds with various doses and different times, the microarray data were first normalized by the median value of all the transcripts per chip. Subsequently each transcript data of all the samples at a time point (24 h or 48 h) for a compound was normalized by the mean value of the transcript of the time matched control samples at the same time point for the same compound. To obtain the comparison of whole genome level expression profiles, we used a total of 22,676 flag filtered transcripts and 40 average conditions to perform a two-way hierarchical clustering to build a condition tree since that data were normalized based on relative control samples, control conditions were excluded in the tree construction. Each dose per time point for one compound was a unique condition, so a total of 40 average unique conditions were in the tree.

### Gene functional analysis and network construction

Significantly regulated probes were employed for one way hierarchical clustering (only cluster genes) or two-way hierarchical clustering (clustering both genes and samples) using GeneSpring 7.0 and/or 10 (Agilent Technologies, Foster City, CA, USA). A Pearson correlation with average linkage was applied for the clustering. Gene functional categories were classified according to Gene Ontology (GO) [70] reference using The Database for Annotation, Visualization and Integrated Discovery (DAVID) [71], [72] and Gofetch [73] tools. A Gene Ontology functional term enrichment p value less than 0.05 was considered significant. Pathway analysis was performed using the Ingenuity canonical pathways analysis tool. Similar to GO analysis, a pathway with an enrichment p value less than 0.05 was considered to be a significantly regulated pathway (Ingenuity Systems, Inc., Redwood City, CA). Gene networks were constructed based on the Ingenuity knowledge base. A score was assigned to a network according to the fit of the original set of significant genes. This score reflects the negative logarithm of the p value that indicates the likelihood of the focus genes in a network being found together due to random chance [74].

### Reverse-transcription quantitative PCR (QRT-PCR)

Two-stage RT-QPCR were performed, 1000 ng of total RNA were first reverse transcribed into cDNA in a 20-µl reaction containing 250 ng random primers and SuperScript™ III reverse transcriptase (Invitrogen) following the manufacture's instruction. The synthesized cDNA was diluted to10 ng/µl as cDNA template. QPCR was performed on an Applied Biosystems Incorporated (ABI, Foster City, CA) Sequence Detector 7900. Each 20-µl reaction was run in duplicate and contained 6 µl (10 ng/µl) of synthesized cDNA templates and 3 ul of nuclease-free water along with 1 ul of TaqMan gene specific assay and 10 ul of 2X TaqMan universal PCR Master Mix (ABI). Cycling parameters were 95°C for 15 min to activate the DNA polymerase, then 40 cycles of 95°C for 15 s and 60°C for 1 min.

### ESI-MS/MS lipid profiling

Lipids were extracted as described [75]. Briefly, homogenized liver tissues were added with 2.5 ml of chloroform/methanol/distilled water (1∶1∶0.5, v/v), and two 0.5-ml chloroform extractions. Combined organic phases were washed with 0.5 ml of KCl (1 m, 1×) and with 0.5 ml of dH_2_0 (2×) and dried with N_2_ gas. Samples were resuspended in 1 ml of chloroform for the subsequent analysis. An automated electrospray ionization-tandem mass spectrometry approach was used for lipidomics study, and data acquisition and analysis were carried out as described previously [19], [76] with minor modifications. Precise amounts of internal standards, obtained and quantified as previously described [19], [20], were added in the following quantities (with some small variation in amounts in different batches of internal standards): 0.60 nmol di12∶0-PC, 0.60 nmol di24∶1-PC, 0.60 nmol 13∶0-lysoPC, 0.60 nmol 19∶0-lysoPC, 0.30 nmol di12∶0-PE, 0.30 nmol di23∶0-PE, 0.30 nmol 14∶0-lysoPE, 0.30 nmol 18∶0-lysoPE, 0.30 nmol 14∶0-lysoPG, 0.30 nmol 18∶0-lysoPG, 0.30 nmol di14∶0-PA, 0.30 nmol di20∶0(phytanoyl)-PA, 0.20 nmol di14∶0-PS, 0.20 nmol di20∶0(phytanoyl)-PS, 0.23 nmol 16∶0-18∶0-PI, 0.16 nmol di18∶0-PI, 2.5 nmol 13∶0 cholesteryl ester (CE), and 2.5 nmol 23∶0 CE. The sample and internal standard mixture was combined with solvents, such that the ratio of chloroform/methanol/300 mM ammonium acetate in water was 300/665/35, and the final volume was 1.2 ml. This mixture for the mass spec was centrifuged for 15 min to pellet particulates before presenting to the autosampler. Unfractionated lipid extracts were introduced by continuous infusion into the ESI source on a triple quadrupole MS/MS (API 4000, Applied Biosystems, Foster City, CA). Samples were introduced using an autosampler (LC Mini PAL, CTC Analytics AG, Zwingen, Switzerland) fitted with the required injection loop for the acquisition time and presented to the ESI needle at 30 µl/min. Sequential precursor and neutral loss scans of the extracts produce a series of spectra with each spectrum revealing a set of lipid species containing a common head group fragment. Lipid species were detected with the following scans: PC, SM, and lysoPC, [M+H]^+^ ions in positive ion mode with Precursor of 184.1 (Pre 184.1); PE and lysoPE, [M+H]^+^ ions in positive ion mode with Neutral Loss of 141.0 (NL 141.0); PI, [M+NH_4_]^+^ in positive ion mode with NL 277.0; PS, [M+NH4]^+^ in positive ion mode with NL 185.0; PA, [M+NH_4_]^+^ in positive ion mode with NL 115.0; CE, [M+NH_4_]^+^ in positive ion mode with Pre 369.3. SM was determined from the same mass spectrum as PC (precursors of m/z 184 in positive mode) [23]–[24] and by comparison with PC internal standards using a molar response factor for SM (in comparison with PC) determined experimentally to be 0.39. The collision gas pressure was set at 2 (arbitrary units). The collision energies, with nitrogen in the collision cell, were +28 V for PE, +40 V for PC (and SM), +25 V for PI, PS and PA, and declustering potentials were +100 V for all lipids except CE, for which the declustering potential was +225 V. Entrance potentials were +15 V for PE, +14 V for PC (and SM), PI, PA, and PS, and+10 V for CE. Exit potential were +11 V for PE, +14 V for PC (and SM), PI, PA, PS, and +10 V for CE. The mass analyzers were adjusted to a resolution of 0.7 u full width at half height. For each spectrum, 9 to 150 continuum scans were averaged in multiple channel analyzer (MCA) mode. The source temperature (heated nebulizer) was 100°C, the interface heater was on, +5.5 kV or −4.5 kV were applied to the electrospray capillary, the curtain gas was set at 20 (arbitrary units), and the two ion source gases were set at 45 (arbitrary units).

### Lipid profile data analysis and statistics

The background of each spectrum was subtracted, the data were smoothed, and peak areas integrated using a custom script and Applied Biosystems Analyst software (Foster City, CA, USA). The lipids in each class were quantified in comparison to the two internal standards of that class. The first and typically every 11^th^ set of mass spectra were acquired on the internal standard mixture only. Peaks corresponding to the target lipids in these spectra were identified and molar amounts calculated in comparison to the internal standards on the same lipid class. To correct for chemical or instrumental noise in the samples, the molar amount of each lipid metabolite detected in the “internal standards only” spectra was subtracted from the molar amount of each metabolite calculated in each set of sample spectra. Finally, the data were corrected for the fraction of the sample analyzed and normalized to the sample “dry weights” to produce data in the units nmol/mg. To compare the different lipids species between groups, data was first filtered based on processed signal in which more than 70% of samples have at least 0.002 nmol/mg for any given lipid species. Otherwise a lipid species was filtered out. Statistic analyses were conducted by One-Way ANOVA across all doses including vehicle controls for a compound. The false discovery rate less than 0.05 after Benjamine-Hochberg correction was taken as the cut off level for significance. A Tukey based post hoc test was applied to identify differentiated lipid species by comparing different groups.

## Supporting Information

Table S1 A table (supplementary table 1) providing a list of genes that were significantly regulated by 2,4DNT at 24 h.
(0.97 MB XLSX)

Table S2 A table (supplementary table 2) providing a list of genes that were significantly regulated by 2,4DNT at 48 h.
(0.04 MB XLSX)

Table S3 A table (supplementary table 3) providing a list of genes that were significantly regulated by 2,6DNT at 24 h.
(0.44 MB XLSX)

Table S4 A table (supplementary table 4) providing a list of genes that were significantly regulated by 2,4DNT at 48 h.
(0.46 MB XLSX)

Table S5 A table (supplementary table 5) providing a list of genes that were significantly regulated by 2ADNT at 24 h.
(0.02 MB XLSX)

Table S6 A table (supplementary table 6) providing a list of genes that were significantly regulated by 2ADNT at 48 h.
(0.02 MB XLSX)

Table S7 A table (supplementary table 7) providing a list of genes that were significantly regulated by 4ADNT at 24 h.
(0.20 MB XLSX)

Table S8 A table (supplementary table 8) providing a list of genes that were significantly regulated by 4ADNT at 48 h.
(0.02 MB XLSX)

Table S9 A table (supplementary table 9) providing a list of genes that were significantly regulated by TNT at 24 h.
(0.02 MB XLSX)

Table S10 A table (supplementary table 10) providing a list of genes that were significantly regulated by TNT at 48 h.
(0.10 MB XLSX)

Table S11 A table (supplementary table 11) providing a list of genes that were commonly significantly regulated by the 5 chemicals.
(0.03 MB XLSX)

Table S12 A table (supplementary table 12) providing a list of genes in the stress and toxicity pathway that were used for QRT-PCR.
(0.03 MB XLSX)

Table S13 A table (supplementary table 13) providing a list of lipid species that were measured in the project.
(0.02 MB XLSX)

File S1 A PDF including supplementary tables 1, 2, 3, 14, 15. Supplementary Figures 1–5.
(0.82 MB DOC)
