# Supplementary material for: Analysis of Common and Specific Mechanisms of Liver Function Affected by Nitrotoluene Compounds
Source: PLoS One. 2011 Feb 8;6(2):e14662. doi: 10.1371/journal.pone.0014662 (PMC3035612; doi:10.1371/journal.pone.0014662)
Supplement: File S1 — A PDF including supplementary tables 1, 2, 3, 14, 15. Supplementary Figures 1– 5. (0.82 MB DOC) [file pone.0014662.s014.doc]

**Supplementary tables**

Supplementary Table 1. Analytical chemistry measurements in blood of nitroaromatic exposed animals after 24h. Numbers represent the concentration per animal. Any concentration <0.200 mg/kg should be considered to be an estimated value because it is below the lowest calibration standard.

|  |  | |
| --- | --- | --- |
| **Compound & Dose Exposed** | TNT | 2ADNT |
| TNT-384 mg/kg | 3.13, 1.32, 0.61, 0.68 | 2.46, 0.94, 1.13, 3.83 |
| TNT-192 mg/kg | 2.85. 3.10 | 4.73, 0.62, 4.61, 0.37 |
| 2ADNT-384 mg/kg |  | 0.07, 0.44 |
| 2ADNT-174 mg/kg |  | 0.63, 1.35, 2.16, 0.66, 0.75 |
| 2ADNT-96 mg/kg |  | 0.05 |

Supplementary Table 2. Analytical chemistry measurements in livers of nitroaromatic exposed animals after 24h. Numbers represent the concentration per animal. Any concentration <0.200 mg/kg should be considered to be an estimated value because it is below the lowest calibration standard.

|  |  | | | | |
| --- | --- | --- | --- | --- | --- |
| **Compound & Dose Exposed** | TNT | 26DANT | 24DANT | 2ADNT | 24DNT |
| TNT-384 mg/kg | 5.65, 0.33, 2.30, 4.73 | 0.64, 1.29 | 0.74, 1.32 |  |  |
| TNT-192 mg/kg | 0.78, 0.09 | 0.21 | 0.5 |  |  |
| TNT-96 mg/kg | 0.42 |  |  |  |  |
| 2ADNT-384 mg/kg |  |  |  | 0.61, 0.40 |  |
| 2ADNT-174 mg/kg |  |  |  | 0.71, 0.21, 0.2 |  |
| 2ADNT-87 mg/kg |  |  |  | 0.37 |  |
| 24DNT-398 mg/kg |  |  |  |  | 0.75 |

Supplementary Table 3 Analytical chemistry measurements in livers of nitroaromatic exposed animals after 48h

| **Compound & Dose Exposed** | 4ADNT | 2ADNT |
| --- | --- | --- |
| TNT-384 mg/kg | 0.05, 0.10 | 0.18, 0.20 |
| TNT-192 mg/kg | 0.04 | 0.11, 0.07 |
| TNT-96 mg/kg |  | 0.07 |
| TNT-48 mg/kg |  | 0.05 |

Supplementary Table 14 Common top significant canonical pathways regulated by all the compounds

| Pathways | Comp-ounds | -Log(P-value) | Most significantly regulated genes |
| --- | --- | --- | --- |
| Aryl Hydrocarbon Receptor Signaling | 2,4DNT | 5.04 | TP53, NFkB, ALDH1B1, GSTM1, GSTM5, MED1,NQO1, ALDH1L1, MDM2,BAX,CCND1,SMARCA4, JUN,GSTM2,NR0B2, FASN, NFIA,CDKN1A,RARB,GSTM4, HSP90AA1,NFE2L2,ALDH6A1,HSPB1 |
|  | 2,6DNT | 1.41 | TP53, GSTM3 (includes EG:2947), NQO1,BAX,SMARCA4,GSTM2,ALDH1L2, GSTM3 (includes EG:14864),ALDH1A3, FASN,CDKN1A, GSTM4, HSP90AA1,NFE2L2, ALDH7A1 |
|  | 2ADNT | 10.5 | ALDH1B1,GSTP1,CYP1A1,ALDH1A1,CYP1A2,HSP90AB1,NQO1,CDKN1A,  HSP90AA1,HSPB1 |
|  | 4ADNT | 6.63 | TP53,ALDH1B1,GSTP1,NQO1,ALDH1L1,SMARCA4,ALDH1A1,HSP90AB1,NR0B2, MGST2,GSTM3 (includes EG:14864),CDK4,NFIA,GSTM4, HSP90AA1, IL1B,NFIB, NCOR2,ATR (includes EG:545),MCM7,HSPB1 |
|  | TNT | 3.82 | GSTM1,GSTT1,GSTP1,ALDH1A1,MGST2,GSTM3 (includes EG:14864),NQO1, GSTM4,ALDH1L1,ALDH3B1,CCND1 |
| NRF2-mediated Oxidative Stress Response | 2,4DNT | 3.84 | AKR7A3,USP14,GSTM5,DNAJA4,HRAS,GCLC,KRAS,CLPP (includes EG:8192),HMOX1,GSTM2,JUN,GSTM4,JUND,FMO1,DNAJB1,FKBP5,NFE2L2,CBR1, GSTM1,NQO1,HERPUD1,TXNRD1,STIP1,DNAJB11,DNAJC7 |
|  | 2,6DNT | 3.14 | AKR7A3,DNAJC17,USP14,GSTM3 (includes EG:2947),CREBBP, NQO1, HRAS, DNAJC13, GCLC,DNAJC10,TXNRD1,MAFG,GSTM2,GSTM3 (includes EG:14864), MAP3K7,STIP1,GSTM4,PIK3R2,FKBP5,NFE2L2,CBR1,EPHX1 |
|  | 2ADNT | 1.64 | AKR7A3,GSTP1,NQO1 |
|  | 4ADNT | 8.61 | AKR7A2,RAF1,AKR7A3,GSTP1,USP14,HSPB8,GCLC,PRKCZ,MAFG,HMOX1,GSTM3 (includes EG:14864),KEAP1,GSTM4,PRKCE, DNAJB1,CBR1,PRKCA, NQO1, MAP3K1,TXNRD1,BACH1,DNAJC11,GSR, MGST2,STIP1, SQSTM1, EPHX1 |
|  | TNT | 6.17 | AKR7A2,AKR7A3,GSTM1,GSTP1,USP14,NQO1,ACTG1,GSR,GSTT1,AKR1A1, ERP29,MGST2,MAP2K2,GSTM3 (includes EG:14864),GSTM4,EPHX1 |
| LPS/IL-1 Mediated Inhibition of RXR Function | 2,4DNT | 2.88 | ALDH1B1,GSTM1,ACSL3,LIPC,GSTM5,ABCB11,ALDH1L1,ABCA1,SLC27A4, TRAF2, AOB,GSTM2,JUN,NR0B2,SREBF1,ACSL5,GSTM4,CPT2,ACSL4,XPO1, FMO1, ABCC3,ACSL1, ALDH6A1 |
|  | 2,6DNT | 2.30 | SULT1C2,ABCG5,LIPC,SLC10A1,GSTM3 (includes EG:2947),MGMT,ACOX1, ABCB11,CYP3A5,GSTM2,ALDH1L2,GSTM3 (includes EG:14864),MAP3K7, ALDH1A3, ACSL5,GSTM4,XPO1,SULT1C3,ABCC3,CYP4A11,ALDH7A1 |
|  | 2ADNT | 3.30 | ALDH1B1,GSTP1,ALDH1A1,SULT1A1,ABCC3 |
|  | 4ADNT | 2.65 | ALDH1B1,GSTP1,CPT1A,MAP3K1,ALDH1L1,CES2 (includes EG:8824),SLC27A4,ALDH1A1,MGST2,NR0B2,GSTM3 (includes EG:14864),NR1I3,GSTM4,XPO1,IL1B,ABCC3,ABCC4 |
|  | TNT | 5.66 | ECSIT,GSTM1,GSTP1,ALDH1L1,SLCO1A2,GSTT1,FABP2,ALDH1A1,MGST2,GSTM3 (includes EG:14864),SULT1A1,CYP7A1, GSTM4,ALDH3B1,ABCC3, SULT1B1 |
| Pyruvate Metabolism | 2,4DNT | 2.13 | AKR7A3,ALDH1B1,ACSL3,ACAT2,PC,NUDT7,ACSS2,ACYP1,ACACA,ACSL1, BCKDHB |
|  | 2,6DNT | 3.16 | AKR7A3,ACAT2,ALDH1A3,ME3,ACSS2,ACYP1,ACOT9,PCK1,LDHA,HADHA,ALDH7A1 |
|  | 2ADNT | 2.94 | AKR7A3,ALDH1B1,ALDH1A1 |
|  | 4ADNT | 1.97 | AKR7A2,AKR7A3,ALDH1B1,ALDH1A1,ACAA1,NUDT7,DLAT |
|  | TNT | 4.54 | AKR7A2,TCAG7.1260,AKR7A3,AKR1A1,ALDH1A1,PCK2,ACAA1,HADHA,AKR1B7 |
| Glutathione Metabolism | 2,4DNT | 1.32 | GSTM1,GSTM2,GSTM5,ACSS2,GSTM4,GCLC,IDH3A,GSS |
|  | 2,6DNT | 1.53 | GSTM2,GSTM3 (includes EG:14864),GSTM3 (includes EG:2947),ACSS2, GSTM4, GCLC, IDH3A |
|  | 2ADNT | 1.87 | GSTP1 |
|  | 4ADNT | 5.31 | EARS2,GSR,NADSYN1,CCDC92 (includes EG:80212),GCLC,CAD,GSS |
|  | TNT | 8.31 | GSR,GSTM1,GSTT1,GSTP1,MGST2,GSTM3 (includes EG:14864),GSTM4,IDH2, GPX4, GSTT3,GSS |

| Pathways | Comp-ounds | -Log(P-value) | Most significantly regulated genes |
| --- | --- | --- | --- |
| Xenobiotic Metabolism Signaling | 2,4DNT | 1.11 | ALDH1B1,GSTM1,GSTM5,MED1,PPP2R5B,NQO1,HRAS,GCLC,ALDH1L1,KRAS, PPP2CB,HMOX1,MAOB,GSTM2,NCOA1,GSTM4,HSP90AA1,FMO1,ABCC3,NFE2L2,DNAJC7,ALDH6A1 |
|  | 2,6DNT | 2.1 | SULT1C2,CES2 (includes EG:234671),UGT1A6,HRAS,GCLC,CYP3A5, GSTM2, UGT2B4, ALDH1A3,GSTM3 (includes EG:14864),GSTM4,PIK3R2, NFE2L2,ALDH7A1, MGMT,GSTM3 (includes EG:2947),NQO1,CREBBP,PPP2CB, PPP2R1A,ALDH1L2, PPP2R2B,HSP90AA1,SULT1C3,ABCC3 |
|  | 2ADNT | 10.8 | ALDH1B1,GSTP1,CYP1A1,ALDH1A1,CYP1A2,UGT1A6,HSP90AB1,SULT1A1, NQO1,HSP90AA1,UGT1A3,ABCC3 |
|  | 4ADNT | 5.31 | RAF1,GSTP1,UGT1A6,GCLC,ALDH1L1,CES2 (includes EG:8824),PRKCZ,HMOX1, ALDH1A1,HSP90AB1,GSTM3 (includes EG:14864),KEAP1,NR1I3,GSTM4, PRKCE,PRKCA,ALDH1B1,NQO1,MAP3K1,UGT1A3,PPP2R1A,MGST2,HSP90AA1, IL1B,NCOR2,ABCC3 |
|  | TNT | 5.05 | GSTM1,GSTP1,UGT1A6,NQO1,ALDH1L1,UGT1A3,UGT1A1,GSTT1,ALDH1A1,MGST2,MAP2K2,GSTM3 (includes EG:14864),SULT1A1, GSTM4,ALDH3B1,ABCC3, SULT1B1 |
| Metabolism of Xenobiotics by Cytochrome P450 | 2,4DNT | 0.683 | GSTM1,CYP4F8,GSTM2,GSTM5,CYP2C7,GSTM4,ALDH1L1,CYP2D6,CYP2D12, ADHFE1,CYP51A1 |
|  | 2,6DNT | 2.83 | CYP2D26,UGT1A6,CYP2C70,GSTM3 (includes EG:2947),CYP2C44,CYP3A5, CYP3A43,GSTM2,UGT2B4,ALDH1A3,GSTM3 (includes EG:14864),GSTM4, CYP2J9, AKR1C4,ADHFE1,EPHX1 |
|  | 2ADNT | 5.32 | GSTP1,CYP1A1,CYP1A2,UGT1A6,UGT1A3 |
|  | 4ADNT | 1.8 | ADH6,GSTP1,MGST2,UGT1A6,GSTM3 (includes EG:14864),GSTM4, ALDH1L1, UGT1A3,GSTT3,EPHX1 |
|  | TNT | 7.26 | GSTM1,GSTP1,UGT1A6,ALDH1L1,UGT1A3,GSTT3,UGT1A1,GSTT1,CYP3A43,MGST2,GSTM3 (includes EG:14864),GSTM4,ALDH3B1,EPHX1 |
| Propanoate Metabolism | 2,4DNT | 4.62 | ALDH1B1,ACSL3,ACAT2,SUCLG2,ACADSB,MUT,DHCR24,PCCA,ACSS2,MCEE,ACACA,EHHADH,ACSL1,ALDH6A1 |
|  | 2,6DNT | 4.86 | ACAT2,SUCLG2,PCCB,GCDH,PCCA,DHCR24,ALDH1A3,ACSS2,EHHADH,SDS,ACADM,LDHA,HADHA,ALDH7A1 |
|  | 2ADNT | 1.81 | ALDH1B1,ALDH1A1 |
|  | 4ADNT | 0.409 | ALDH1B1,ALDH1A1,ACAA1 |
|  | TNT | 3.05 | ALDH1A1,ACAA1,MLYCD,ACAD9,HADHA,ACADS |
| Fatty Acid Metabolism | 2,4DNT | 2.47 | ALDH1B1,ACSL3,ACAT2,ACADSB,CYP2D6,CYP2D12,SLC27A4,CYP4F8,ACSL5,CPT2,CYP2C7,ACSL4,EHHADH,ADHFE1,ACSL1,HADH,CYP51A1 |
|  | 2,6DNT | 3.27 | CYP2D26,ACAT2,CYP2C70,ACOX1,CYP2C44,CYP3A5,GCDH,CYP3A43,ALDH1A3,ACSL5,CYP2J9,EHHADH,ACADM,SDS,ADHFE1,HADHA,CYP4A11,ALDH7A1 |
|  | 2ADNT | 3.01 | ALDH1B1,CYP1A1,ALDH1A1,CYP1A2 |
|  | 4ADNT | 0.57 | ADH6,ALDH1B1,ALDH1A1,CPT1A,ACAA1,SLC27A4,ACAA2 |
|  | TNT | 2.3 | CYP3A43,ALDH1A1,ACAA1,ACAD9,ACAA2,DCI,HADHA,ACADS |
| Valine, Leucine and Isoleucine Degradation | 2,4DNT | 4.37 | ALDH1B1,ACAT2,ACADSB,MUT,BCKDHB,PCCA,HIBADH,MCEE,MCCC1, EHHADH,ALDH6A1,HADH,AOX3,MCCC2 |
|  | 2,6DNT | 3.62 | ACAT2,PCCB,GCDH,PCCA,ALDH1A3,HIBADH,MCCC1,EHHADH,SDS,ACADM, HADHA,ALDH7A1,MCCC2 |
|  | 2ADNT | 1.71 | ALDH1B1,ALDH1A1 |
|  | 4ADNT | 0.965 | ALDH1B1,ALDH1A1,BCAT2,ACAA1,ACAA2 |
|  | TNT | 2.77 | ALDH1A1,ACAA1,ACAD9,ACAA2,HADHA,ACADS |

Supplementary Table 15 Common significant canonical pathways

**Figure legends:**

**Supplementary Fig. 1** **Hierarchical clustering of experimental conditions.** A. Experimental conditions were based on averaging samples with various concentrations at the same time point for the same compound. So dose responses were ignored. Total 10 conditions were included for the clustering. B. Experimental conditions were based on averaging all the samples for a compound, no matter which dose and what time were treated. Therefore, total only 5 conditions were included in the clustering. Transcripts used for making these conditional trees were selected based on filtering on flags. Only a transcript that was presented in at least 50% samples was chosen for clustering. Pearson correlation algorithm with average linkage was conducted to calculate distances between conditions.

**Supplementary Fig. 2 Quantitative real-time RT-PCR (QRT-PCR) verification of microarray gene expression using genes associated with oxidative stress.**  96 genes that are responsible for oxidative stress were selected to perform real time quantitative QRT-PCR. The samples of controls or treated with 2,4DNT at 199mg/ml for 24 h (A) or 48 h (B) were used for PCR tests. Four biological replicates were used for QRT-PCR. A fold change was calculated by comparing exposed versus controls at each time point. The up-regulated and down-regulated genes are presented as positive number and negative numbers respectively. Only the genes whose expression was significantly changed by at least one method are shown here. All the genes were consistently significantly regulated using both methods. The significance was determined using a p value <0.05 of statistical t-test. A regression coefficient between the two approaches for 24 h (C) and 48 h (D) were calculated.

**Supplementary Fig. 3 Quantitative real-time RT-PCR (QRT-PCR) verification of microarray gene expression using some selected genes.** Some genes were selected to validate the microarray results. Both QRT-PCR and microarray results of the genes PPARα, TF, APOB, Fabp2, GSTM7-7 or GSTM4, Aldh1a1, aHG were presented. The experimental design was the same as the microarray experiment. The expression value for a given gene was represented as a log2 ratio of exposed versus respective control RNA. For a gene, the upper panel is RT-PCR results, the lower panel is microarray results. The numbers 1, 2, 3, 4 represent lowest, second lowest, second highest and highest dose respectively for one of these compound for the microarray experiment design. Bars represent the standard errors for the average log2 ratios of biological replicates.

**Supplementary Fig. 4 The correlation between the microarray and RT-PCR data for RT-PCR validated genes.**

**Supplementary Fig. 5 Hypoxia signaling affected by 2,4DNT.** Most of genes in the Hypoxia signaling were significantly upregulated by 2,4 DNT (red highlighted).

**Fig. 1A**

**
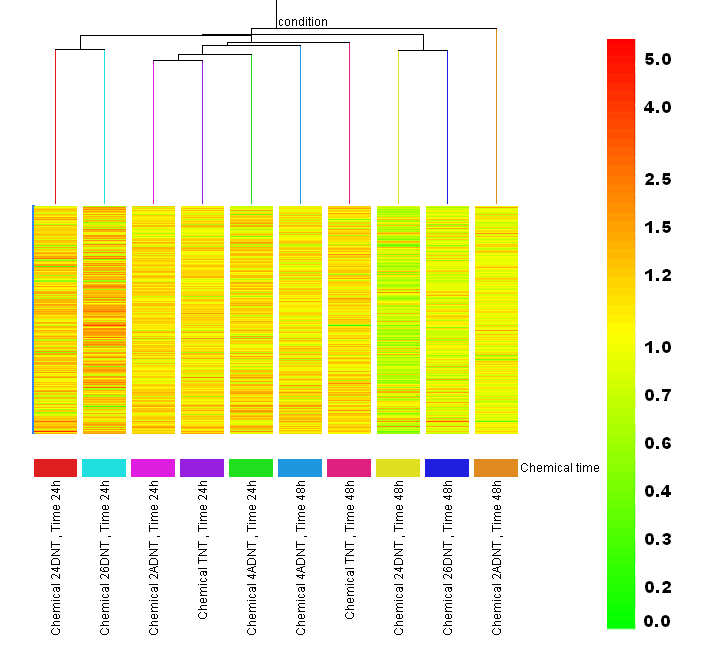
**

**Fig. 1B**

**
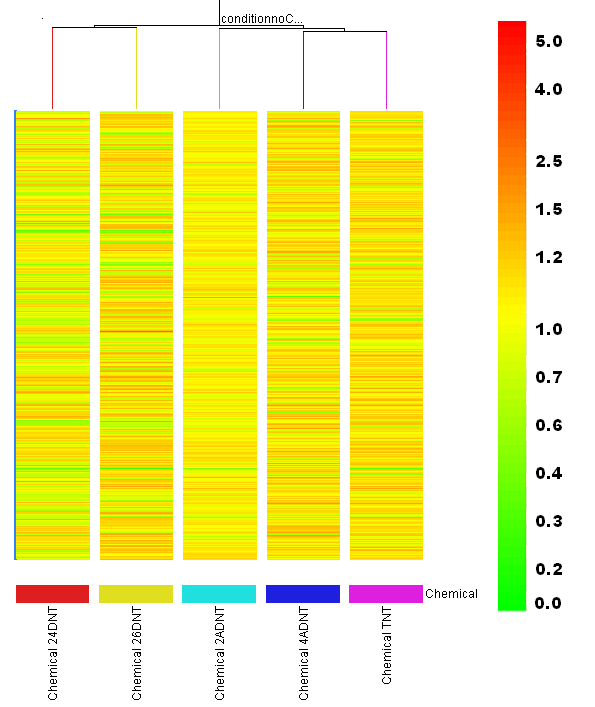
**

**Fig. 2**

**
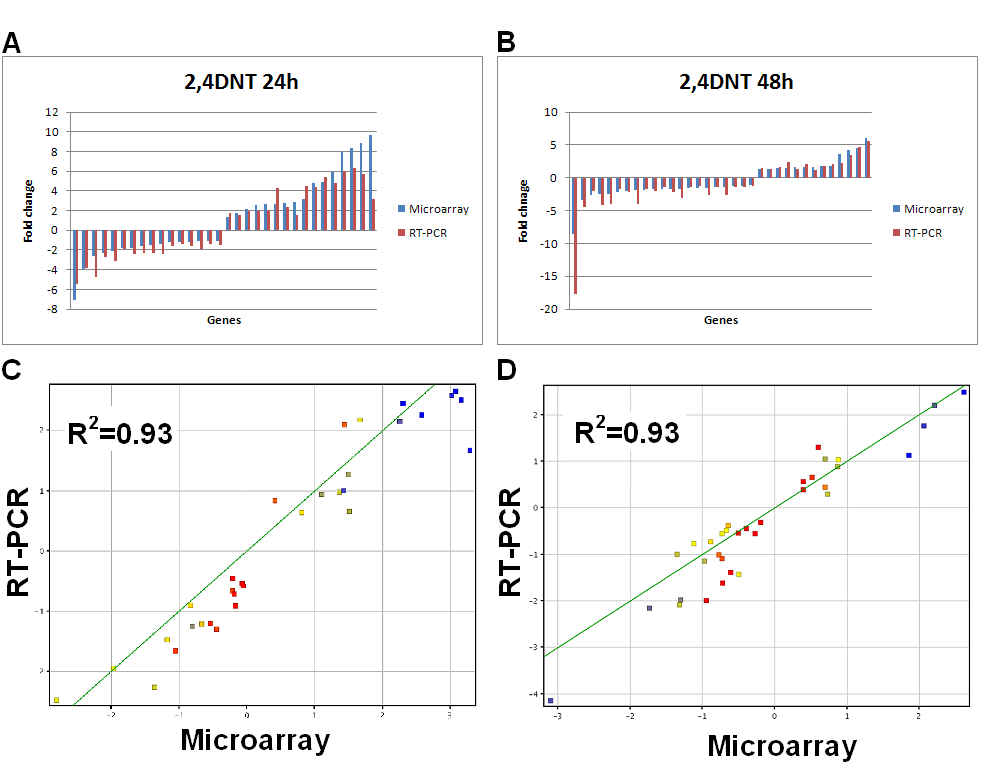
**

**Fig. 3
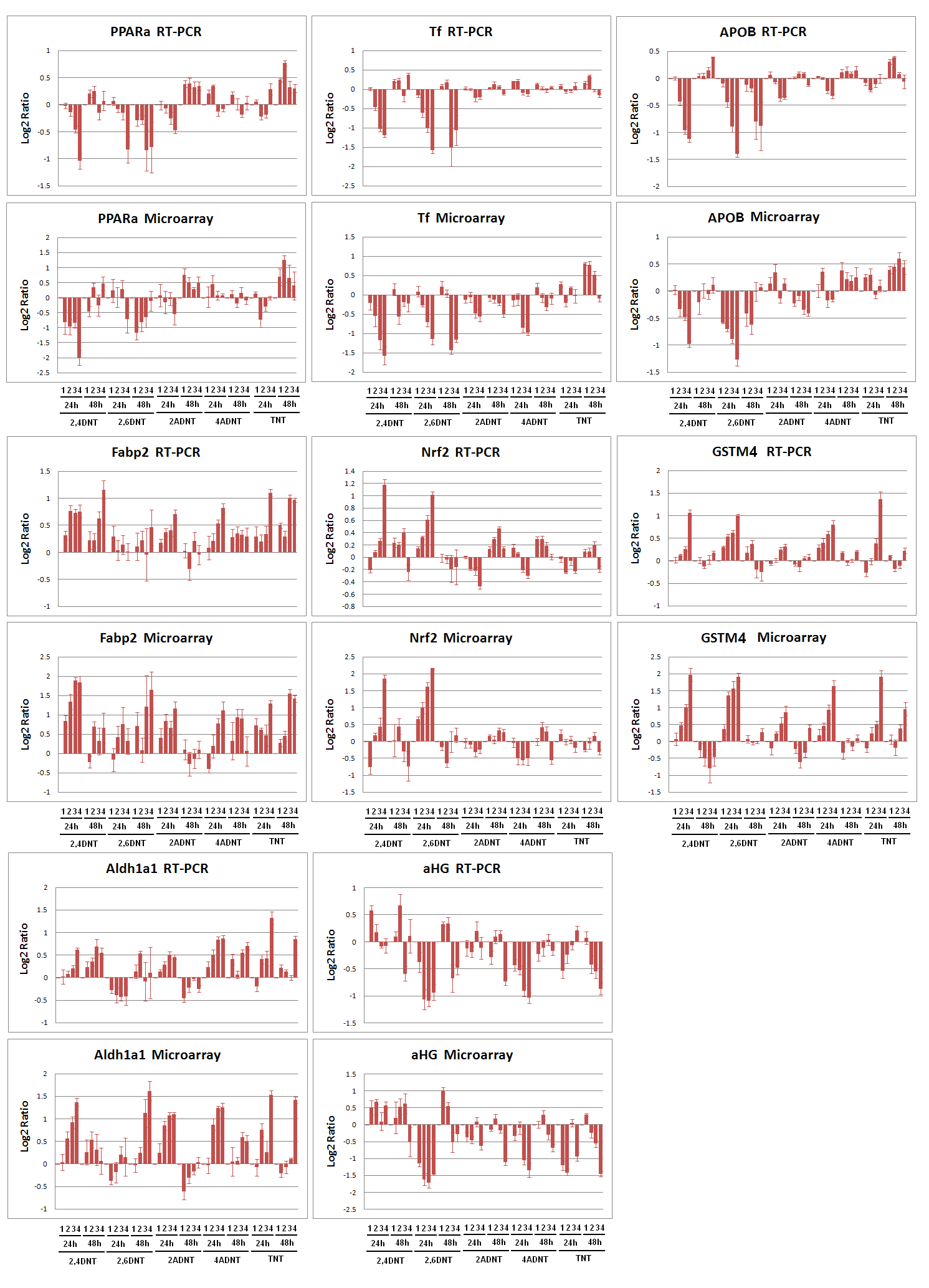
**

**Fig. 4**

**
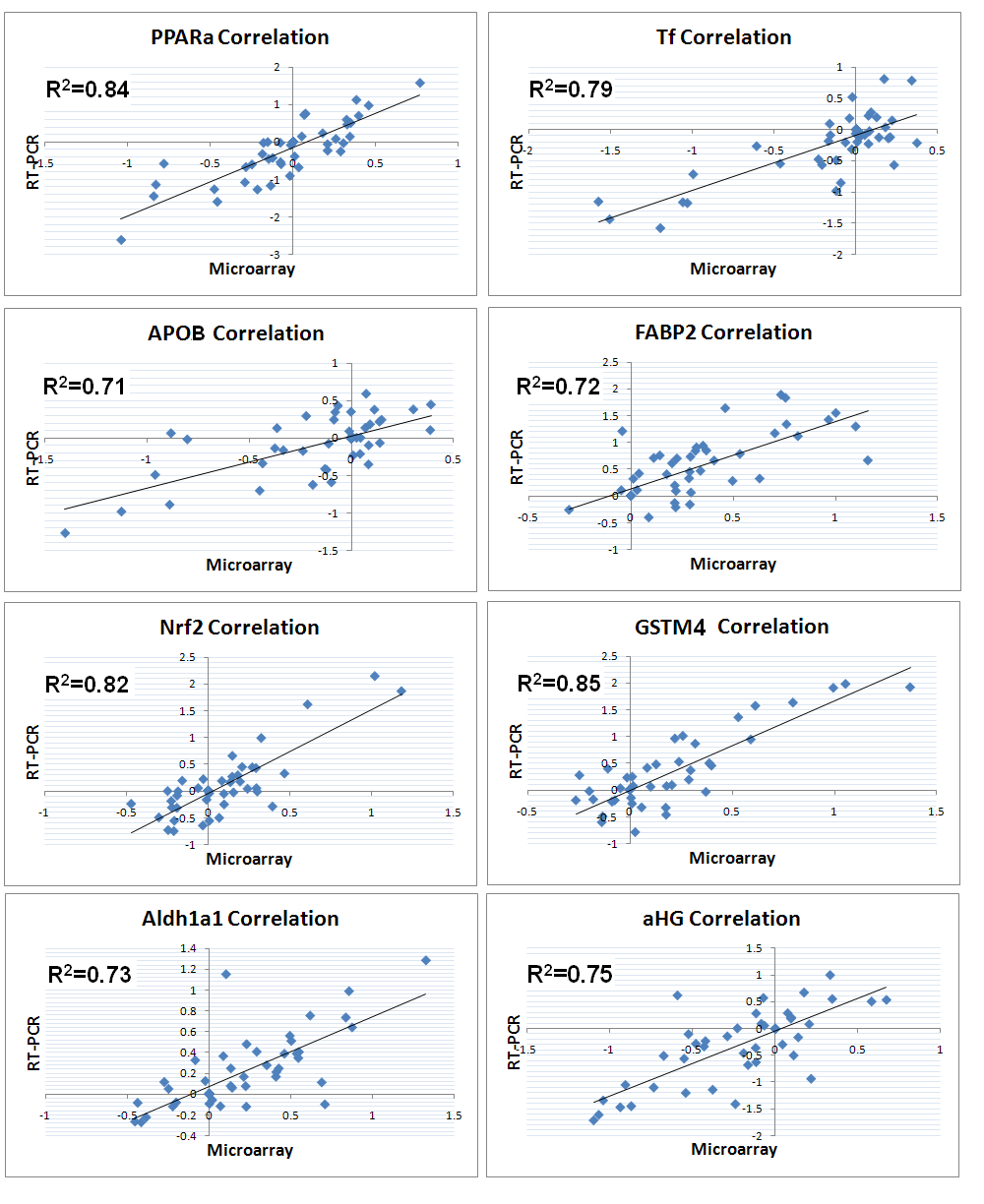
**

**Fig. 5**

**
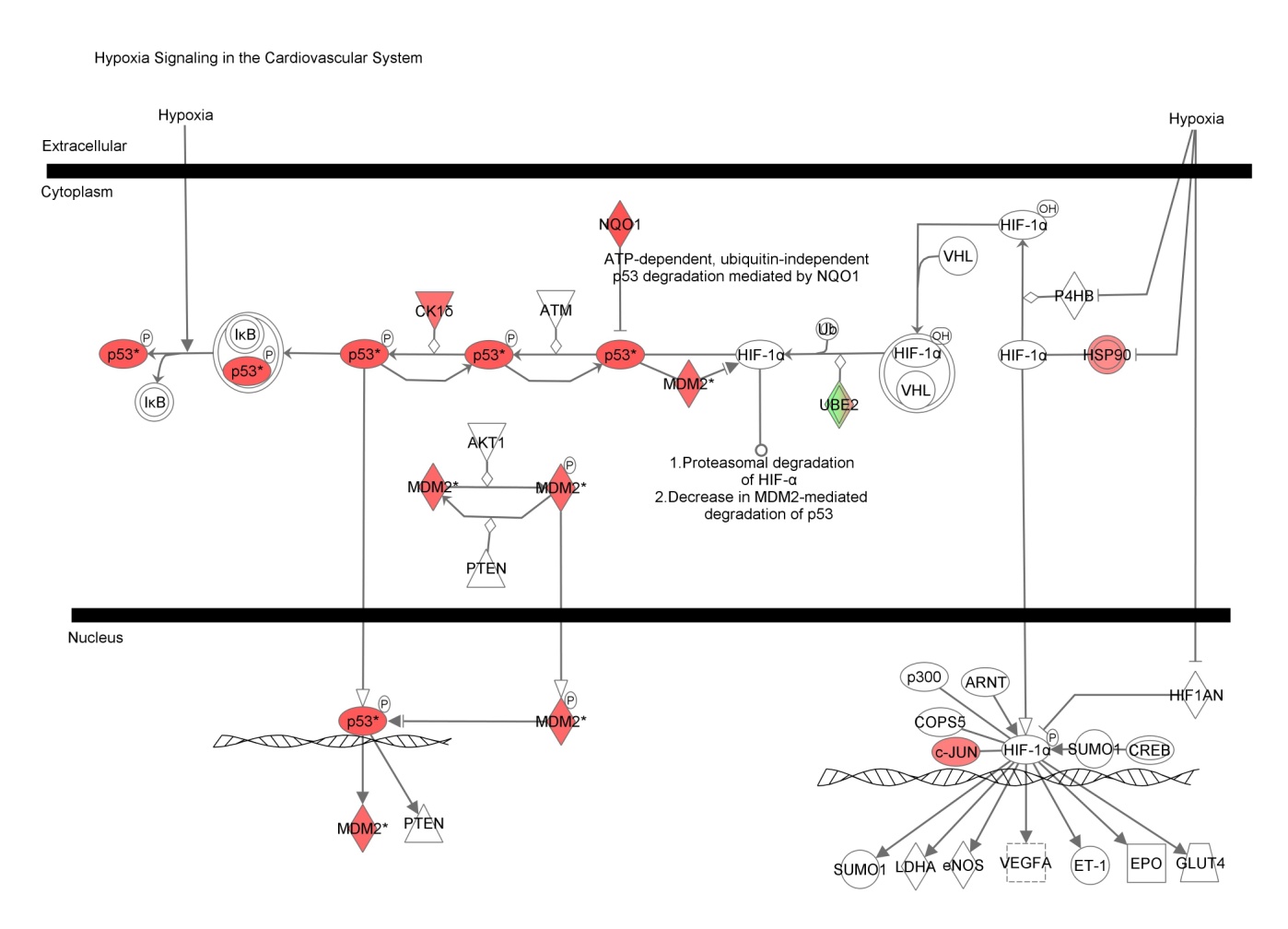
**
